# Psychometric features of the Patient-Centered Communication Scale among Iranian clinical nurses

**DOI:** 10.1371/journal.pone.0358575

**Published:** 2026-09-18

**Authors:** Saeed Ghasempour, Hossein Bagheri, Hamid Sharif-Nia, Zahra Ashrafi, Mohammadjavad Bagherian, Ali Abbasi

**Affiliations:** 1 Student Research Committee, School of Nursing and Midwifery, Shahroud University of Medical Sciences, Shahroud, Iran; 2 Department of Nursing, School of Nursing and Midwifery, Shahroud University of Medical Sciences, Shahroud, Iran; 3 Psychosomatic Research Center, Mazandaran University of Medical Sciences, Sari, Iran; 4 Department of Nursing, Amol Faculty of Nursing and Midwifery, Mazandaran University of Medical Sciences, Sari, Iran; 5 Center for Health Related Social and Behavioral Sciences Research, Shahroud University of Medical Sciences, Shahroud, Iran; School of Nursing Sao Joao de Deus, Evora University, PORTUGAL

## Abstract

**Background:**

Measuring patient-centered communication seems essential from the perspective of nurses as the primary healthcare providers. The Patient-Centered Communication Scale (PCCS) is one of the instruments that makes it possible to achieve this goal. Therefore, since no version apart from the original version of this instrument has been psychometrically tested in other countries, including Iran, this study aimed to culturally adapt and psychometrically evaluate the PCCS in Iranian clinical nurses.

**Methods:**

The current methodological study was conducted in hospitals associated with Shahroud University of Medical Sciences. Content validity was examined through qualitative and quantitative methods by specialists in patient-centered communication and nurse-patient communication. Factor analysis, including exploratory and confirmatory approaches, was employed to determine the factor structure and model fit of the Persian version of the PCCS. The convenience sampling approach enabled the achievement of the aforementioned goal by collecting two independent samples of 150 qualified clinical nurses each, totaling 300 qualified clinical nurses. Internal consistency was evaluated using Cronbach’s alpha and McDonald’s omega coefficients, while external stability was quantified by the intraclass correlation coefficient (ICC).

**Results:**

The expert panel’s comments suggested deleting item 4 due to substantial conceptual overlap with item 5. Exploratory factor analysis revealed a single factor with an eigenvalue of 8.56, explaining 77.78% of the entire variance of the Persian version of this scale. Confirmatory factor analysis also supported the single-factor structure of the Persian version of the mentioned scale. The Cronbach’s alpha and McDonald’s omega coefficients exceeded 0.70, indicating satisfactory internal consistency, while the ICC value exceeded 0.80, showing favorable external stability.

**Conclusion:**

The favorable psychometric features suggest the use of the Persian version of the mentioned scale to identify factors related to this concept in clinical settings and determine the effectiveness of various interventions to promote this specific type of communication. However, the single-center data collection constrains the generalizability of the findings to clinical nurses employed in medical centers linked to other Iranian universities of medical sciences and non-university facilities, including private medical centers.

## Introduction

Communication is an indispensable component of the nursing profession, facilitating the comprehension, investigation, and attention of the unique requirements of each patient [1]. This key component is essential for establishing therapeutic relationships with patients and their families, as well as fostering collaborative relationships with other healthcare providers (HCPs) [2]. However, nurses frequently encounter difficulties in communicating with patients, their families, and other HCPs. These challenges reduce the standard of nursing care and increase the likelihood of medication errors, which could result in undesirable consequences or even mortality [3]. Improved communication skills among nurses have also been associated in several studies with lower medical errors, outstanding nursing care, greater clinical performance, increased self-efficacy, stronger organizational commitment, and enhanced job satisfaction [4–6].

Therefore, nurses should communicate in a goal-oriented manner in their clinical practice, focusing on promoting the physical and mental health of patients. This approach is called patient-centered communication [7], also known as person-centered communication or client-centered communication [8]. Joo *et al.* (2024) defined patient-centered communication as a series of actions designed to empower patients and their families. This encompasses providing sufficient information, emotional support, empathetic expression, actively reflecting their values and preferences in decision-making, and engaging in treatment-related decisions [9]. In other words, patient-centered communication denotes a process that promotes the active involvement of patients and their families in decision-making on their care requirements [8].

Patient-centered communication cultivates trust and mutual respect in the care process, enhancing practices that correspond with the needs, concerns, and preferences of patients and caregivers [2]. This concept is crucial in achieving desirable health outcomes, as it reflects longstanding nursing values that prioritize individualized care [2]. The goal of patient-centered communication is to prevent illness and promote patient well-being through their active participation in treatment and decision-making, all based on respect for patients and their families [10]. In contrast, ineffective nurse-patient communication undermines patients’ trust in HCPs, which can lead to them withholding important information that requires immediate intervention [11]. This ineffective communication can also lead to increased length of stay, patient dissatisfaction, and waste of resources [12], as well as reduced quality of nursing care and increased patient stress. It can also make patients feel insecure and uninformed, leading them to view HCPs as inexperienced or incompetent [13]. Consequently, it is important to have a comprehensive, valid, and reliable instrument to assess patient-centered communication from the viewpoint of nurses as primary HCPs and to identify its associated factors in clinical settings.

The Global Interpersonal Communication Competence Scale (GICCS) is commonly used to assess the communication skills of clinical nurses [14], despite not being specifically designed for nurses in clinical settings [9]. Alshammari *et al.* (2021) also conducted a study to determine the psychometric properties of the Arabic version of the Patient-Centered Communication Instrument (PCCI). This 36-item instrument, with satisfactory validity and reliability, measures six subscales: (a) exchanging information, (b) fostering healthy relationships, (c) making decisions, (d) responding to emotions, (e) enabling patient self-management, and (f) managing uncertainty from the perspectives of adults with cancer [15]. Similarly, Demiris *et al.* (2023) evaluated the validity and reliability of the English version of the Caregiver-Centered Communication Questionnaire (CCCQ). This 30-item questionnaire not only has desirable psychometric properties but also measures five subscales: (a) exchange of information, (b) fostering health relationships, (c) recognizing and responding to emotions, (d) managing care, and (e) decision-making from the perspective of family caregivers of patients hospitalized in hospice care [16].

The aforementioned instruments, while possessing suitable psychometric indicators and evaluating multiple dimensions of patient-centered communication, were specifically designed and psychometrically validated to measure this concept from the viewpoint of patients and their families [15,16]. However, patient-centered communication is an interactive concept, and assessing it solely from the perspective of patients and their families may not provide a comprehensive understanding of how this approach is realized in clinical practice. Nurses, who are the primary HCPs and have the most frequent and continuous interactions with patients and their families throughout this process, play a fundamental role in transforming the principles of patient-centered communication into everyday practical behaviors. Therefore, their perspective can provide unique information about communication behaviors, clinical decision-making processes, contextual factors, and organizational conditions that facilitate or inhibit this specific type of communication. On the other hand, evaluating this concept from the perspective of nurses may lead to identifying training needs, designing quality improvement programs, and developing effective interventions to improve patient-centered communication practices. For example, nurses can provide valuable information about how to apply communication strategies in clinical settings, how to address the needs, preferences, and concerns of patients and their families, how to facilitate their participation in the care process, and how to collaborate with other members of the treatment team. Such information can help identify factors that influence the achievement of patient-centered communication and better understand the mechanisms that facilitate or inhibit it, aspects that cannot be assessed solely from the perspective of patients and their families. Therefore, assessing patient-centered communication from the perspective of nurses does not replace assessing it from the perspective of patients and their families, but rather complements it. This approach contributes to a more multidimensional understanding of this concept in clinical settings [9].

In this regard, Joo *et al.* (2024) conducted a study with the aim of designing and validating the Patient-Centered Communication Scale (PCCS) in Korean clinical nurses. After conceptualizing patient-centered communication, the mentioned study designed the initial items of this scale through a literature review and online interviews with 10 qualified clinical nurses. 51 items were selected as the initial items of this scale. Following the removal, integration, and modification of some items in the content validity stage, a 31-item scale was obtained. Then, the remaining items were perceptually tested in terms of understandability, completion time, and layout appropriateness by 10 qualified clinical nurses. The construct validity of the mentioned scale was assessed using exploratory factor analysis (EFA) (on 175 qualified clinical nurses) and confirmatory factor analysis (CFA) (on 150 qualified clinical nurses). Finally, 12 items remained, covering three factors: (a) information sharing (5 items), (b) patient-as-person (4 items), and (c) therapeutic alliance (3 items). The positive and significant correlation between the PCCS and the GICCS also suggested its desirable convergent validity. Its reliability was also reported to be acceptable by the internal consistency method through Cronbach’s alpha coefficient for the aforementioned factors, as well as the entire scale [9].

Accordingly, Joo *et al.* (2024) introduced a valid and reliable instrument that assesses patient-centered communication as a three-dimensional construct by implementing a step-by-step and systematic approach [9]. On the other hand, no study has yet addressed the cultural adaptation and psychometric evaluation of the PCCS in clinical nurses from other societies. Only Amin *et al.* (2025) in a study that examined the mediating role of moral distress in the relationship between patient-centered communication and palliative care competence among Egyptian oncology nurses, despite not evaluating the psychometric properties of this scale in the aforementioned society, referred to the PCCS as a three-dimensional scale and used its total score in the analyses [17]. However, no other evidence was found regarding the factor structure and model fit of the original version of this scale in clinical nurses from other societies. Therefore, the present study, while translating and culturally adapting, first examines the three-dimensional model presented by the initial study of scale design, including information sharing, patient-as-person, and therapeutic alliance through CFA. If the presented three-dimensional model is rejected, then the factor structure and model fit of the Persian version of the PCCS are determined through exploratory and confirmatory approaches of factor analysis, respectively.

## Materials and methods

### Design and participants

The present methodological study utilized a cross-sectional approach to initially translate and culturally adapt the PCCS for Iranian clinical nurses. Subsequently, the validity (in four forms: face, content, construct, and convergent) and reliability (using three approaches: internal consistency, construct reliability, and external stability) of the Persian version of this scale were evaluated in the aforementioned population.

To achieve the study objective, two independent samples of 150 individuals each, totaling 300 clinical nurses from hospitals associated with Shahroud University of Medical Sciences (Imam Hossein and Bahar Hospitals), were chosen through a convenience sampling approach adhering to specified inclusion and exclusion criteria from May 10, 2025, to October 20, 2025.

### Inclusion and exclusion criteria

The inclusion criteria required a bachelor of science in nursing (BSN) or higher, as well as a minimum of one year of full-time clinical experience. A minimum of one year of full-time clinical experience not only provides sufficient exposure to care situations and patient-centered interactions but also increases the likelihood of accurately comprehending the items based on actual clinical experience. On the other hand, a BSN or higher, as the minimum level of professional education in many nursing systems, including Iran, facilitates understanding and interpretation of the content of the items. These criteria were selected in line with previous psychometric studies in Iranian clinical nurses [18–20]. Conversely, the exclusion criteria included any interdepartmental transfers within the previous month.

### Data gathering

Following the acquisition of the required approvals from the Vice Chancellor for Research and Technology at Shahroud University of Medical Sciences, essential connections were established with the respected officials of Imam Hossein and Bahar Hospitals. Subsequently, the principal investigator was present in the study environment, introduced himself to each participant, and explained the study’s objectives. Oral and written informed consent was secured from the participants to partake in the study. Finally, all participants were provided with the data collection instruments, including the demographic characteristics checklist and the PCCS, to complete in their spare time.

#### Demographic characteristics checklist.

This checklist includes information on age, gender, marital status, education level, work experience, department of practice, employment status, and income adequacy. Income adequacy evaluated the sufficiency of monthly income to cover living expenses using a three-point Likert scale: below average, average, and above average. The department of practice included internal wards (chemotherapy, internal medicine, gastroenterology, cardiology, post cardiac care unit [Post CCU], and infectious diseases); surgical wards (surgery, urology, and orthopedics); critical care units (dialysis, intensive care unit [ICU], and cardiac care unit [CCU]); emergency rooms; pediatrics and neonatology, as well as others. It is worth noting that each clinical nurse was assigned to work in only one of the aforementioned departments.

#### Patient-Centered Communication Scale.

This scale was developed and validated by Joo *et al.* (2024) to assess patient-centered communication skills in nurses. The PCCS consists of 12 items that assess three factors: (a) information sharing (items 1–5), (b) patient-as-person (items 6–9), and (c) therapeutic alliance (items 10–12). Each item is scored on a five-point Likert scale, with responses ranging from strongly disagree (1 point) to strongly agree (5 points). The numerical scores range from 12 (indicating the lowest patient-centered communication skills) to 60 (indicating the highest patient-centered communication skills), with higher scores representing more patient-centered communication skills in nurses and vice versa [9].

### Translation and cultural adaptation

The translation and cultural adaptation of the PCCS were based on the model established by Wild *et al.* (2005), which included ten steps [21].

#### Step 1: Preparation.

After corresponding with Dr. Yang, the original designer of the scale, permission for translation and psychometric testing into Persian was obtained via email.

#### Step 2: Forward translation.

Two expert translators, fully proficient in English and Persian language and culture, independently translated the original version of the scale into Persian.

#### Step 3: Reconciliation.

The study team evaluated the two resulting Persian versions and consolidated them into a single version.

#### Step 4: Backward translation.

Two experienced translators, uninvolved in the preliminary translation and unaware of the current study process, independently translated the merged Persian version into English.

#### Step 5: Review of backward translations.

The study team compared the two English versions obtained and consolidated them into a single version. Finally, the merged English version was sent to the original scale designer for approval.

#### Step 6: Harmonization.

The final version from the previous step was compared to the original scale to identify and eliminate any linguistic problems and vocabulary differences. This confirmed alignment between the two versions.

#### Step 7: Cognitive debriefing.

Ten qualified clinical nurses were given the final version to identify any possible ambiguities or errors.

#### Step 8: Review of cognitive debriefing.

The study team reviewed the feedback from the nurses and discussed any issues raised. Necessary changes were then made to the final version.

#### Step 9: Proofreading.

A Persian language and literature expert corrected the final version for writing, grammar, and other errors.

#### Step 10: Final report.

After documenting all steps, the final version was used to assess validity and reliability [22,23].

### Face validity

The face validity of the PCCS was evaluated using both qualitative and quantitative methods.

#### Qualitative face validity assessment.

In the qualitative method, face-to-face interviews were conducted with ten qualified clinical nurses who had a diverse range of demographic characteristics. These interviews aimed to evaluate each item in terms of difficulty, relevance, and ambiguity [24,25].

#### Quantitative face validity assessment.

In the quantitative method, a five-point Likert scale was utilized, with responses varying from “completely understandable” (5 points) to “not understandable at all” (1 point). Ten qualified clinical nurses rated each item using this Likert scale. The impact score (IS) for each item was subsequently calculated using the following formula:


$$\begin{array}{c} 𝐈𝐒=𝐅𝐫𝐞𝐪𝐮𝐞𝐧𝐜𝐲\;(\%)\;\times \;𝐂𝐨𝐦𝐩𝐫𝐞𝐡𝐞𝐧𝐬𝐢𝐯𝐞𝐧𝐞𝐬𝐬 \end{array}$$ (1)

Frequency (%) denotes the proportion of respondents assigning a score of 4 or 5 to an item, whereas Comprehensiveness reflects the average score of that item on the Likert scale. Items with an IS above 1.50 are retained for further analysis, whereas those with an IS below 1.50 are revised but not eliminated [25,26].

### Content validity

Similar to face validity, both qualitative and quantitative methods were employed to evaluate the content validity of the PCCS.

#### Qualitative content validity assessment.

In the qualitative method, the mentioned scale was provided to 10 nursing faculty members and specialists in patient-centered communication and nurse-patient communication. This group consisted of two nursing professors, two associate professors of nursing, two assistant professors of nursing, two clinical psychologists, and two instrument development specialists. Their feedback on grammar, sentence structure, item placement, and scoring accuracy was then collected [27,28].

#### Quantitative content validity assessment.

In the quantitative method, the content validity ratio (CVR), the content validity index (CVI) at both item and scale levels, and the modified Kappa statistic (K^*^) were calculated.

To determine the CVR, which assesses the necessity of items in the scale, a group of 10 specialists in the field of patient-centered communication and nurse-patient communication evaluated each item using a three-point Likert scale (unnecessary = 1 point, useful but unnecessary = 2 points, necessary = 3 points). The CVR was then calculated using the following formula:


$$\begin{array}{c} 𝐂𝐕𝐑=\frac{𝐧𝐄\;-\;\frac{\;𝐍\;}{2}}{\frac{\;𝐍\;}{2}} \end{array}$$ (2)

nE denotes the count of specialists who regard the item in question as essential, whereas N represents the total number of specialists. The Lawshe table facilitates result interpretation, with CVR values exceeding 0.62 signifying the importance of items within the scale [29].

To ascertain the item-level content validity index (I-CVI), which measures the relationship of items to the scale, another group of 10 specialists in the aforementioned fields evaluated each item on a four-point scale: not pertinent = 1 point, somewhat pertinent = 2 points, pertinent but requires amendment = 3 points, and completely pertinent = 4 points. The I-CVI for each item was determined by dividing the number of specialists who rated the item as 3 or 4 by the total number of specialists [30,31]. The scale-level content validity index/average (S-CVI/Ave) is calculated by averaging the I-CVI scores. This is done by dividing the sum of the I-CVI scores by the total number of items [32].

I-CVI values less than 0.70 are considered unacceptable, values between 0.70 and 0.78 are deemed questionable, and values greater than 0.79 are considered acceptable. Therefore, unacceptable items are removed, questionable items are modified, and acceptable items are retained [30]. Additionally, S-CVI/Ave values greater than 0.90 are considered desirable [32].

The K^*^ was calculated for each item to reduce the likelihood of random agreement. To do this, the probability of chance agreement (P_c_) must first be determined using the following formula:


$$\begin{array}{c} {𝐏}_{𝐜}=\left[\frac{𝐍!}{\;𝐀!(𝐍-𝐀)!\;}\right]\times \;0.5^{𝐍} \end{array}$$ (3)

N denotes the total count of specialists, whereas A signifies the number of specialists who evaluated the item with a rating of 3 or 4. Subsequent to calculating the P_c_, the K^*^ was ascertained utilizing the following formula:


$$\begin{array}{c} {𝐊}^{*}=\frac{\;𝐈-𝐂𝐕𝐈\;-\;{𝐏}_{𝐜}\;}{1\;-\;{𝐏}_{𝐜}} \end{array}$$ (4)

Therefore, K^*^ values less than 60.00% are considered poor, 60.00% to 74.00% are considered good, and greater than 75.00% are considered excellent [33].

### Demographic characteristics of participants

Qualitative demographic characteristics were described separately for the first and second independent samples through frequency and percentage. These samples were formally compared in terms of these variables by means of the chi-square and Fisher’s exact tests. Quantitative demographic characteristics were also reported separately for the first and second independent samples through mean and standard deviation (SD). These samples were formally compared in terms of these variables by means of the independent t-test.

### Construct validity

Factor analysis was used to assess construct validity. The analytical strategy followed a sequential approach. First, the originally proposed three-factor model of the PCCS [9] was tested using CFA on the first independent sample (n = 150). Because this a priori model exhibited inadequate fit, we proceeded with an exploratory approach. Accordingly, EFA was performed on the first independent sample (n = 150) to uncover the underlying factor structure. Finally, the factor structure emerging from the EFA was subjected to a subsequent CFA on the second independent sample (n = 150) to evaluate its model fit.

#### Exploratory factor analysis.

The first independent sample of 150 eligible clinical nurses underwent EFA using the maximum likelihood (ML) estimation. Kaiser-Meyer-Olkin (KMO) values surpassing 0.80, along with the significance of Bartlett’s test of sphericity (*P <* 0.001), confirmed the adequacy of the sampling [34,35]. Factors were extracted according to (a) eigenvalues surpassing one, (b) communalities exceeding 0.20, and (c) factor loadings exceeding 0.30 [35]. The eigenvalue of each factor was determined by aggregating the squares of the factor loadings of all associated items. This eigenvalue was subsequently split by the total number of items to ascertain the percentage of the entire variance it explains [36]. These analyses were conducted using SPSS software version 24.

#### Confirmatory factor analysis.

The second independent sample of another 150 eligible clinical nurses underwent CFA to evaluate the factor structure identified from maximum likelihood exploratory factor analysis (MLEFA). The ML estimation was applied for all CFA models. Modification indices (MI) were also examined to detect local misspecifications and to guide any theoretically justified residual covariances. Values less than 3 for the χ^2^ divided by degrees of freedom (χ^2^/df) and values less than 0.08 for the root mean square error of approximation (RMSEA) as absolute fit indices; values greater than 0.90 for the comparative fit index (CFI) and Tucker-Lewis index (TLI) as comparative fit indices; and values greater than 0.50 for the parsimony normed fit index (PNFI) and parsimony comparative fit index (PCFI) as parsimonious fit indices confirmed the goodness-of-fit of the aforementioned model [24,37].

In addition, a competing three-factor model, as originally proposed by Joo *et al.* (2024), was also tested using the same sample [9]. The fit of the two models was compared using the chi-square difference test (Δχ^2^) and by examining the change in comparative fit indices (ΔCFI≥0.01 as an indication of non-negligible difference), as well as the Akaike information criterion (AIC) and Bayesian information criterion (BIC) for model parsimony.

During the initial CFA of the unidimensional model, MI were inspected to identify local misspecifications. Residual covariances were considered for addition only when the MI exceeded 10 and the correlation was theoretically justifiable based on content overlap or similar wording (e.g., shared method effects due to common thematic content). The fit of the initial model (without correlated residuals) and the final model (with theoretically supported residual correlations) were compared. These analyses were conducted using AMOS software version 24.

### Convergent validity

The method established by Fornell and Larcker (1981) provides an assessment of convergent validity by calculating composite reliability (CR) and average variance extracted (AVE) [38]. Acceptable convergent validity for PCCS requires meeting one of the following conditions: (a) CR values exceeding 0.70, (b) CR values surpassing AVE, or (c) AVE values exceeding 0.50 [38].

### Reliability

Initially, internal consistency was investigated by computing Cronbach’s alpha and McDonald’s omega coefficients. Subsequently, construct reliability was assessed by calculating CR and maximal reliability (MaxR). Values of Cronbach’s alpha and McDonald’s omega coefficients surpassing 0.70 indicated desirable internal consistency of the PCCS. Conversely, values of CR and MaxR surpassing 0.70 also confirmed the acceptable construct reliability of this scale [34,39].

Finally, the test-retest method, following the calculation of the intraclass correlation coefficient (ICC) with a two-way mixed effects model, examined the external stability. Completion of the PCCS in two stages, with a fourteen-day interval, by 25 eligible clinical nurses made it possible to achieve this goal. The mentioned time interval was chosen in accordance with the methodological recommendations of psychometric studies. While it is long enough to reduce the likelihood of participants recalling previous responses, it is short enough to prevent real changes in perceptions of patient-centered communication [40,41]. Participants in this phase were also randomly selected with maximum diversity in demographic characteristics. Their mean age and work experience were reported as 37.36 ± 8.51 and 13.42 ± 8.69, respectively. Additionally, 21 (84.00%) were female and 4 (16.00%) were male. Furthermore, 7 (28.00%) worked in internal wards, 10 (40.00%) in surgical wards, 3 (12.00%) in critical care units, and 5 (20.00%) in emergency rooms. ICC values greater than 0.80 also confirmed the satisfactory external stability of this scale [24,42].

### Overall scores of the Patient-Centered Communication Scale

Descriptive indices such as mean, median, SD, and range (minimum and maximum) for the overall scores of the single-factor structure of this scale were calculated separately for the first and second independent samples.

### Normal distribution and outlier values

Skewness values spanning −3.00 to +3.00 and kurtosis values spanning −7.00 to +7.00, as well as Mardia coefficient values less than 8.00, indicated no substantial deviation from the univariate and multivariate normal distribution of the items, respectively [35,37]. These assessments were performed to justify the use of the ML estimator in the subsequent factor analyses, as ML estimation is robust to moderate violations of normality but benefits from approximately normal distributions when used with 5-point Likert-type data under adequate sample sizes. Although the items are ordinal, the observed skewness, kurtosis, and Mardia coefficients supported the applicability of ML estimation. Univariate and multivariate outliers were also examined through distribution plots and Mahalanobis distance (*P <* 0.001) [24,35]. No outliers were detected; thus, no sensitivity analysis using robust estimators was required for the present primary analyses.

### Ethical considerations

The Research Ethics Council of Shahroud University of Medical Sciences approved the present study on March 9, 2025. This approval can be viewed with the ethics code IR.SHMU.REC.1403.192 in the Ethics in Biomedical Research System of the Ministry of Health and Medical Education of Iran at https://ethics.research.ac.ir/. Additionally, informed consent was secured from the participating nurses through verbal explanations and written documentation when explaining and presenting the objectives of the study. The authors also ensured adherence to the principles of the Committee on Publication Ethics (COPE) when publishing the study results.

## Results

### Face validity

The qualitative examination of face validity demonstrated the clarity, appropriateness, and importance of all items. In the quantitative examination of face validity, the IS of items ranged from 3.36 to 4.70. With a threshold value of 1.50, no items required modification or revision.

### Content validity

The qualitative examination of content validity resulted in minor changes to some items. In the quantitative examination of content validity, the CVR values for items 6 and 10 were found to be below the threshold value of 0.62. The I-CVI value for item 10 ranged from 0.70 to 0.78, with a corresponding K^*^ value between 60.00% and 74.00%. As a result, these two items were reviewed and revised accordingly. Additionally, the expert panel’s comments suggested deleting item 4 (*“Describe the medical treatment/procedure or nursing intervention”*) due to substantial conceptual overlap with item 5 (*“Describe the reason for the necessity of medical treatment/procedure or nursing intervention”*). Because in the Iranian healthcare system, both items reflect a common aspect of patient-centered communication and rarely occur independently of each other. The phrase “to the patient or their family” was added to item 5, and “about the patient or their family” was added to item 10. Accordingly, item 5 was revised from *“Describe the reason for the necessity of medical treatment/procedures or nursing intervention”* (original version) to *“Describe the reason for the necessity of medical treatment/procedures or nursing intervention to the patient or their family”* (Persian version). Furthermore, item 10 was changed from *“Communicate and share my negative and positive emotions while providing care with my fellows and/or medical staff (e.g., physician, fellow nurse, etc.)”* (original version) to *“Communicate and share my negative and positive emotions about the patient or their family while providing care with my fellows and/or medical staff (e.g., physician, fellow nurse, etc.)”* (Persian version). The S-CVI/Ave was also calculated as 0.94, which is considered excellent.

### Demographic characteristics of participants

Nurses with official employment status (69.33% for the first sample and 77.33% for the second sample) and a BSN (88.00% for the first sample and 82.00% for the second sample) constituted the majority of the participants in the first and second independent samples. The mean of the participants’ work experience in the first and second independent samples was also 12.51 ± 7.78 and 12.09 ± 7.56, respectively. The two samples were also homogeneous in terms of all demographic characteristics (*P >* 0.05). Other demographic characteristics of the participating nurses are presented in Table 1, separated by the first and second independent samples.

**Table 1:** Demographic characteristics of participants by the first and second independent samples.

Variable type		First sample		Second sample		Test statistics	P
Qualitative variables		n	%	n	%		
Gender	Female	116	77.33	115	76.67	0.019	0.891^a^
	Male	34	22.67	35	23.33		
Marital status	Married	107	71.33	106	70.67	1.654	0.704^b^
	Single	39	26.00	38	25.33		
	Divorced	2	1.33	5	3.33		
	Widow	2	1.33	1	0.67		
Education level	BSN	132	88.00	123	82.00	2.118	0.146^a^
	MSN	18	12.00	27	18.00		
Department of practice	Internal wards	40	26.67	43	28.67	0.510	0.992^a^
	Surgical wards	23	15.33	21	14.00		
	Critical care units	42	28.00	39	26.00		
	Emergency rooms	32	21.33	32	21.33		
	Pediatrics and neonatology	9	6.00	11	7.33		
	Others	4	2.67	4	2.67		
Employment status	Officially	104	69.33	116	77.33	2.455	0.117^a^
	Non-officially	46	30.67	34	22.67		
Income adequacy	Below average	120	80.00	122	81.33	0.978	0.884^b^
	Average	29	19.33	28	18.67		
	Above average	1	0.67	150	100.00		
**Quantitative variables**		**M**	**SD**	**M**	**SD**	**Test statistics**	**P**
Age (year)		36.92	8.51	36.21	7.87	0.747	0.456^c^
Work experience (year)		12.51	7.78	12.09	7.56	0.474	0.636^c^

**n =** Frequency; **% =** Percent; **BSN =** Bachelor of Science in Nursing; **MSN =** Master of Science in Nursing; **M =** Mean; **SD =** Standard Deviation; **P =** P-value; ^**a**^ **=** Chi-Square Test; ^**b**^ **=** Fisher’s Exact Test; ^**c**^ **=** Independent t-Test.

### Construct validity

Before conducting EFA, the three‑factor model proposed by Joo *et al.* (2024) was tested using CFA on the first independent sample (n = 150). This initial model yielded the following fit indices: χ^2^(41)=210.091, *P <* 0.001, χ^2^/df = 5.12, RMSEA = 0.11, CFI = 0.86, TLI = 0.88, PNFI = 0.74, and PCFI = 0.75. Additionally, the correlations among the three factors exceeded 0.85, indicating poor discriminant validity. Based on these inadequacies—particularly the unsatisfactory RMSEA and the high interfactor correlations—the original three‑factor structure was rejected. Consequently, we proceeded with EFA on the first sample to explore a better‑fitting structure.

The KMO value of 0.97 and Bartlett’s sphericity test (χ^2^ = 4292.43, df = 55.00, *P <* 0.001) indicated the adequacy of the sampling. Conversely, skewness values (±3.00) and kurtosis values (±7.00), along with the Mardia coefficient values (<8.00), respectively, demonstrated no substantial deviation from the univariate and multivariate normal distribution of the items. Following this, EFA using the ML approach extracted only one factor with an eigenvalue of 8.56, covering 77.78% of the entire variance of the Persian version of the PCCS. The lowest and highest commonality values and factor loadings were attributed to items 6 (h^2^ = 0.59 and factor loading = 0.77) and 9 (h^2^ = 0.92 and factor loading = 0.96), respectively (Table 2).

**Table 2:** Exploratory factor analysis to evaluate the factor structure of the Persian version of the Patient-Centered Communication Scale (n = 150).

Factors	Items	Factor loading	h^2^	λ	% Variance
**Patient-Centered Communication Scale**	Q9. Listen actively to the words of the patient and family members when communicating.	0.96	0.92	8.56	77.78
	Q11. Introduce departments or other organizations that can help the patient or family (e.g., social work team, caregiver association, psychotherapy etc.)	0.93	0.87		
	Q5. Describe the reason for the necessity of medical treatment/procedures or nursing intervention to the patient or their family.	0.93	0.87		
	Q12. Collaborate across disciplines (e.g., physicians, nutritionist, physical therapist, etc.) to establish therapeutic interventions and aid patient and/or family’s understanding.	0.93	0.86		
	Q3. Provide enough time to ask questions.	0.91	0.84		
	Q1. Identify the information patient/families want to know and provide it in a timely manner.	0.91	0.82		
	Q8. Look at the situation from the patient or family’s point of view and communicate with them.	0.87	0.76		
	Q2. Check the patient or family’s level of understanding after providing the information.	0.86	0.73		
	Q7. Encourage the patient or family to express any feelings they may experience related to the illness.	0.82	0.67		
	Q10. Communicate and share my negative and positive emotions about the patient or their family while providing care with my fellows and/or medical staff (e.g., physician, fellow nurse, etc.)	0.80	0.64		
	Q6. Use open-ended questions to allow the patient and family to express their feelings, needs, and opinions freely (e.g., “How are you feeling today?”)	0.77	0.59		

**h**^**2**^=Communalities; **λ**=Eigenvalue.

After the EFA extracted a single factor, this unidimensional model was tested using CFA on the second independent sample (n = 150) and demonstrated excellent fit. To formally compare the retained unidimensional structure with the original three-factor solution, the three-factor model was also re-estimated on the same CFA sample (χ^2^(39)=104.52, *P <* 0.001, χ^2^/df = 2.68, RMSEA = 0.08, CFI = 0.98, TLI = 0.97, PNFI = 0.72, and PCFI = 0.73). Although this three-factor model met some conventional fit thresholds, the Δχ^2^ between the unidimensional and three-factor models was not significant (Δχ^2^(3)=4.21, *P =* 0.24), and ΔCFI was 0.01, which is below the conventional threshold for a meaningful improvement. Because the three-factor model already demonstrated satisfactory fit on the second independent sample, we did not examine its modification indices for residual correlations, as further adjustments were not warranted. Residual covariances were added only to the initially poorly fitting unidimensional model, and the final model comparison was conducted between the well-fitting three-factor model and the revised unidimensional model, using Δχ^2^, ΔCFI, AIC, and BIC as recommended. Moreover, the AIC and BIC values favored the more parsimonious unidimensional model (AIC = 189.63 vs. 195.84; BIC = 253.21 vs. 262.45). Therefore, the unidimensional structure was retained as the final model.

**Fig 1:**
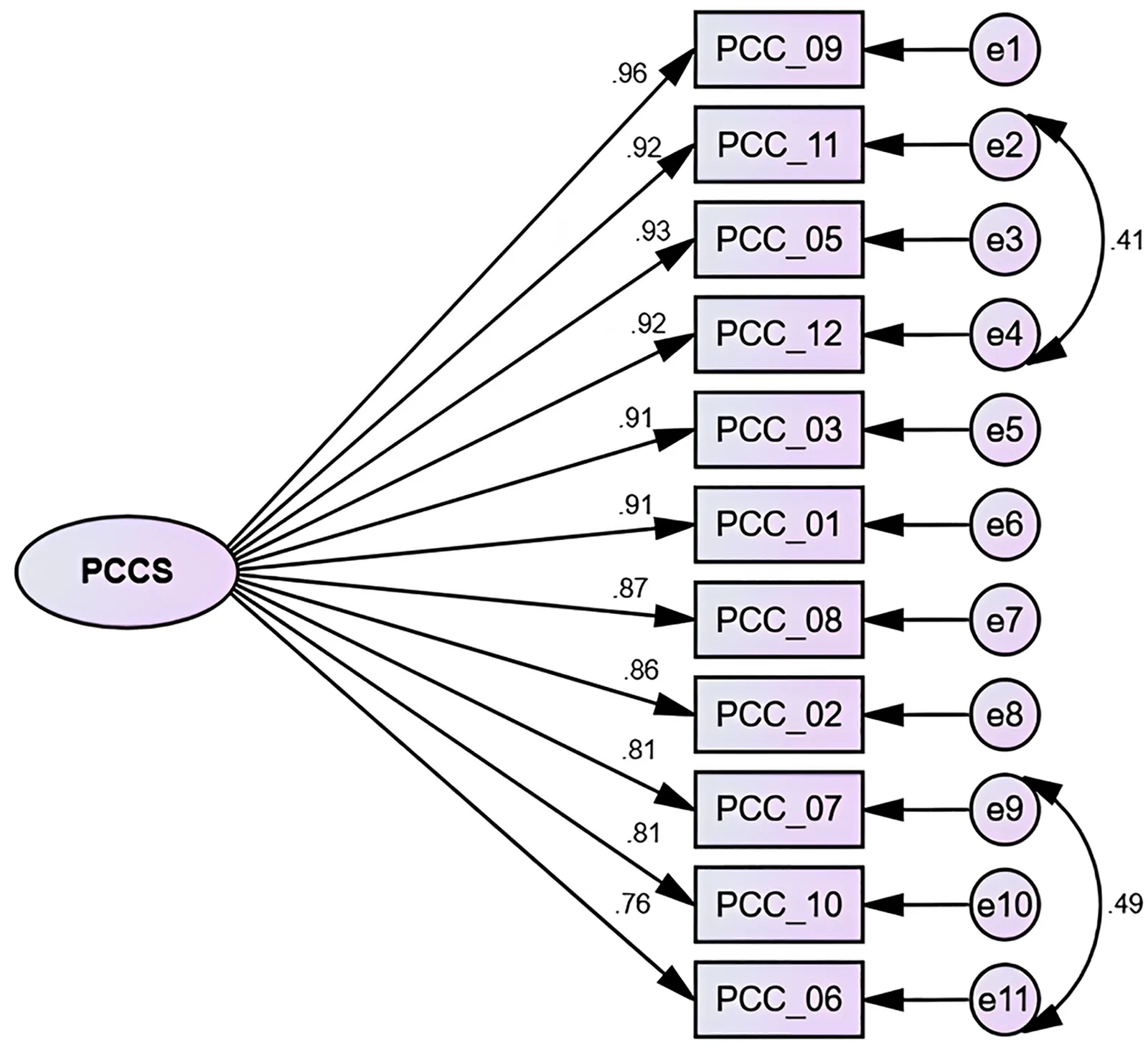
The conclusive model of the Persian version of the Patient-Centered Communication Scale (n = 150).

Before reaching this final model, the initial unidimensional solution without correlated residuals was tested and showed a slightly suboptimal fit (χ^2^(44)=185.24, *P <* 0.001, χ^2^/df = 4.21, RMSEA = 0.12, CFI = 0.89, TLI = 0.87, PNFI = 0.47, and PCFI = 0.48). Inspection of modification indices revealed that adding covariances between the residuals of items 11 and 12 and items 6 and 7 would substantially improve the model. These correlations were theoretically defensible: items 11 (*“Introduce departments or other organizations that can help the patient or family”*) and 12 (*“Collaborate across disciplines to establish therapeutic interventions”*) both concern interdisciplinary coordination and external resource linkage; items 6 (*“Use open‑ended questions to allow the patient and family to express their feelings”*) and 7 (*“Encourage the patient or family to express any feelings related to the illness”*) both focus on eliciting and responding to patients’ emotional expressions. After adding these two residual covariances, the final model achieved the excellent fit (χ^2^(42)=103.63, *P <* 0.001, χ^2^/df = 2.47, RMSEA = 0.07, CFI = 0.99, TLI = 0.98, PNFI = 0.75, and PCFI = 0.75). Table 3 compares the fit of different CFA models, including the three-factor model provided by the initial study of scale design on the first and second independent samples, as well as the single-factor model obtained from the EFA with and without correlated residuals. Fig 1 also displays the conclusive model of the Persian version of the mentioned scale.

**Table 3:** Comparison of the fits of different confirmatory factor analysis models.

Model	χ²	df	P	Parsimonious fit		Comparative fit		Absolute fit	
				χ^2^/df	RMSEA	CFI	TLI	PNFI	PCFI
**Three‑factor** **(On first sample)**	210.091	41	*<*0.001	5.12	0.11	0.86	0.88	0.74	0.75
**Three‑factor** **(On second sample)**	104.52	39	*<*0.001	2.68	0.08	0.98	0.97	0.72	0.73
**One-factor** **(Without correlated residuals)**	185.24	44	*<*0.001	4.21	0.12	0.89	0.87	0.47	0.48
**One-factor** **(With correlated residuals)**	103.63	42	*<*0.001	2.47	0.07	0.99	0.98	0.75	0.75

**df =** degrees of freedom; **P =** P-value; **RMSEA =** Root Mean Square Error of Approximation; **CFI =** Comparative Fit Index; **TLI =** Tucker–Lewis Index; **PNFI =** Parsimony Normed Fit Index; **PCFI =** Parsimony Comparative Fit Index.

### Convergent validity

As indicated in Table 4, the CR value surpasses 0.70, the CR value exceeds the AVE, and the AVE value surpasses 0.50, confirming the convergent validity of the single-factor model of the Persian version of the PCCS. It is worth noting that meeting only one of the aforementioned conditions was sufficient to confirm convergent validity.

**Table 4:** The method established by Fornell and Larcker (1981) for assessing convergent validity, along with reliability indices.

Indices	Unidimensional model	Acceptable value
Composite reliability	0.97	>0.70
Average variance extracted	0.78	>0.50
Maximal reliability	0.98	>0.70
Cronbach’s alpha coefficient	0.93	>0.70
McDonald’s omega coefficient	0.93	>0.70
Intraclass correlation coefficient	0.87	>0.80

### Reliability

The Cronbach’s alpha and McDonald’s omega coefficients, CR, and MaxR values of the Persian version of the PCCS were calculated as 0.93, 0.93, 0.97, and 0.98, respectively (Table 4). Therefore, satisfactory construct reliability was achieved with CR and MaxR values exceeding 0.70, and desirable internal consistency was achieved with Cronbach’s alpha and McDonald’s omega coefficients surpassing 0.70.

Finally, the ICC value of the Persian version of the PCCS was reported as 0.87 (Table 4). Therefore, acceptable external stability was achieved with an ICC value exceeding 0.80.

### Overall scores of the Patient-Centered Communication Scale

Descriptive indices for the overall scores of the single-factor structure of this scale were obtained in the first independent sample: Mean = 37.21, Median = 43.50, SD = 14.83, and Range = 44 (11–55), as well as in the second independent sample: Mean = 36.65, Median = 43.00, SD = 14.87, and Range = 44 (11–55).

## Discussion

This study sought to determine the psychometric features of the PCCS among Iranian clinical nurses. Reviewing the literature about the assessment of the psychometric features of the mentioned scale revealed that the present study is the inaugural evaluation of its validity and reliability across other societies, including Iran. An exploratory approach was used to evaluate the factor structure of the Persian version of the PCCS, which identified only one factor that explained 77.78% of the entire variance of the mentioned scale. A confirmatory approach was also used to evaluate the model fit of the Persian version of the PCCS, showing the goodness of fit of the single-factor model of this scale. Joo *et al.* (2024) also used factor analysis, both exploratory and confirmatory, to assess the construct validity of the original version of the PCCS. Accordingly, EFA extracted three factors, namely (a) information sharing, (b) patient-as-person, and (c) therapeutic alliance, which explained 59.00% of the entire variance of the original version of this scale. CFA also supported the goodness of fit of the three-factor structure of the original version of the mentioned scale [9].

Accordingly, the original version includes 12 items that measure patient-centered communication as a three-dimensional construct [9], while the Persian version includes 11 items that assess this concept as a unidimensional construct. Therefore, the deletion of item 4 (*“Describe the medical treatment/procedure or nursing intervention”*) in the content validity stage is one of the most important differences between this study and the initial study of scale design. Although the original version considered item 4 (what is the medical treatment/procedure or nursing intervention) as a distinct component from item 5 (why is the medical treatment/procedure or nursing intervention), the expert panel in the present study reported significant conceptual overlap between the two items. It should be noted that the conceptual overlap observed between these two items is not due to their simplification or homogenization in the process of translation and cultural adaptation, because in all stages of the model proposed by Wild *et al.* (2005), an attempt was made to maintain the conceptual distinction between what and why. This is while, from their perspective, in everyday nurse-patient interactions, explaining the type and nature of care or treatment is usually accompanied by explaining its cause and necessity. In fact, these two communication behaviors rarely occur independently of each other in such a way that their practical separation seems difficult in many clinical situations. As a result, both items were considered to reflect a common aspect of patient-centered communication, namely, providing information and explaining care or treatment to the patient or their families.

This perception is also consistent with the literature on patient-centered communication, in which information transfer, patient education, and the creation of a shared understanding of the care or treatment situation are considered interconnected components of an integrated communication process [43,44]. On the other hand, in cultural adaptation and psychometric evaluation studies, respondents’ and professionals’ perceptions of the content of the items may be influenced by professional, cultural, and organizational contexts. Therefore, the observation of overlap between items that were considered distinct in the original version does not necessarily mean a change or weakening of the theoretical concepts of the construct; rather, it may reflect how those concepts are expressed and perceived in a different cultural context. In such circumstances, their modification or deletion is considered an acceptable course of action [45]. However, the two items are not entirely identical from a theoretical perspective, as one emphasizes explaining “what is done” and the other emphasizes explaining “why it is done”. Therefore, since maintaining the two mentioned items and evaluating their performance in later stages of psychometric testing could provide more evidence about the degree of differentiation of these two concepts, it is suggested that future research examine this issue in independent samples and in different contexts.

Despite using the same approaches and achieving favorable construct validity results, what distinguishes these two versions from each other is the three-factor nature of the original version of the scale in Korean clinical nurses and the single-factor nature of the Persian version of the scale among Iranian clinical nurses. Conversely, to further validate the factor structure, the resulting unidimensional model was formally compared with the original three-dimensional model. Although both models showed adequate fit, the chi-square difference test (Δχ²) was not significant, and the parsimonious indices (AIC and BIC) clearly supported the unidimensional model. This strengthened the decision to use the unidimensional model for the Persian version. It also indicated that the items were integrated into an overall structure of this concept rather than separate subdomains of patient-centered communication. The difference in the dimensionality of the original and Persian versions of the PCCS can be assessed in many ways, and making a definitive decision about it is not so simple. However, a review of the observed and unobserved factors to which this difference can be attributed is not without merit.

Despite the almost identical inclusion and exclusion criteria in this study and the original study of scale design, the work experience of the participants in the present study (12.30 ± 7.66) was reported as almost twice the work experience of the participants in the original study of scale design (6.25 ± 3.55). However, the two populations did not differ significantly in terms of gender (majority female), level of education (majority BSN), department of practice (majority internal medicine), or age (mean age between 30 and 40 years). Therefore, some apparent differences between the work experience of the participants in the two studies may explain the difference in the dimensionality of the two versions. Other possible reasons for this discrepancy include personal or behavioral differences between Korean and Iranian clinical nurses, as well as the different conditions prevailing in the healthcare systems of the two countries. These factors play a key role in conceptualizing a specific type of communication, namely patient-centered communication, from the viewpoint of nurses as primary HCPs. Moreover, nurses’ favorable attitudes and perceptions are shaped by a combination of organizational factors such as adequate staffing, managerial support, and interprofessional relationships, which may vary considerably across different healthcare systems and cultural contexts [46]. A literature-based analysis of patient-centered communication in interactions between nurses and patients categorized the obstacles to effective communication into four distinct groups: (a) institutional barriers, also known as healthcare system-related barriers, such as deficiency of nursing personnel and excessive workload; (b) communication barriers, such as language differences between patients and HCPs and poor communication skills; (c) environmental barriers, such as noisy environments and untidy and crowded departments; and (d) personal or behavioral barriers, such as demographic characteristics and different cultural backgrounds, beliefs, and worldviews about health and illness [2]. On the other hand, the decision to delete an item at the content validity stage or the use of the English version provided by the original designer as the basis for translation may have contributed to the aforementioned difference to some extent. Although the translation and cultural adaptation process was carried out according to standard guidelines, the possible influence of these factors cannot be completely ruled out. However, examining the possible reasons raised in different societies with various characteristics not only covers institutional, communication, environmental, and personal or behavioral differences as much as possible, but also helps to better understand the origin of the differences in the factor structure of the original and Persian versions of the PCCS.

Beyond comparing the factor structure, examining the position of the PCCS among existing instruments also helps better interpret the results obtained. Although several instruments have been developed to assess patient-centered communication, most of them assess the experience or perception of patients and family caregivers of the quality of communication with HCPs. For example, the PCCI, which has undergone psychometric assessment by Alshammari *et al.* (2021), measures the aforementioned concept from the perspective of adults with cancer [15]. Similarly, the CCCQ, whose validity and reliability have been evaluated by Demiris *et al.* (2023), measures this specific type of communication from the perspective of family caregivers of patients hospitalized in a hospice [16]. These instruments are valuable because they reflect the direct experience or perception of healthcare recipients, whether patients or family caregivers, but they are not designed to measure clinical nurses’ communication skills from their own perspective. In contrast, the PCCS assesses this construct from the perspective of nurses as the primary HCPs and can be particularly useful for identifying training needs or planning interventions to improve communication skills. In addition, the aforementioned translation and validation studies assessed the factor structure only with a confirmatory approach and did not conduct factor analysis with an exploratory approach. However, the present study assessed the construct validity by simultaneously conducting exploratory and confirmatory approaches, while randomly assigning participants to two independent samples. This approach not only fully complies with the rigorous requirements of psychometric assessment but also minimizes overfitting and improves generalizability [37,47,48]. However, the results of the aforementioned instruments may be affected by various biases such as group conformity or social desirability, like other self-report instruments. Therefore, the simultaneous use of instruments based on the perspectives of clinical nurses and those of patients or family caregivers in future research could provide a more comprehensive and realistic assessment of patient-centered communication in clinical settings.

Although Joo *et al.* (2024) suggested the desirable convergent validity of the original version of the PCCS by evaluating its correlation with the GICCS (r = 0.68, *P <* 0.001) [9], the present study exhibited satisfactory convergent validity of the Persian version of the PCCS using the method established by Fornell and Larcker (1981) (CR > 0.70 or CR > AVE or AVE > 0.50). Therefore, despite the different approaches used, both the original and Persian versions of this scale had acceptable convergent validity.

This study confirmed the internal consistency, construct reliability, and external stability of the Persian version of the PCCS. In contrast, Joo *et al.* (2024) only reported the internal consistency of the original version of the PCCS. The Cronbach’s alpha coefficient values for the entire scale and the factors of (a) information sharing, (b) patient-as-person, and (c) therapeutic alliance were 0.84, 0.77, 0.72, and 0.60, respectively [9]. Calculating McDonald’s omega coefficient alongside Cronbach’s alpha coefficient to assess internal consistency, as it is less sensitive to sample size and does not require the assumption of tau equivalence [49,50]; determining construct reliability with two CR and MaxR indices; and assessing external stability by calculating ICC, as it is an integral part of the reliability analysis process [51], distinguish the present study from the initial study of scale design. This study not only addressed the limitations of the initial study of scale design but also showed satisfactory reliability for the Persian version of the mentioned scale in various forms such as internal consistency, construct reliability, and external stability.

After deleting item 4 during the content validity stage, the Persian version of the PCCS now consists of 11 items that assess patient-centered communication as a unidimensional construct. These items are rated using a five-point Likert scale, spanning strongly disagree (1 point) to strongly agree (5 points). Scores can differ from 11 (representing the lowest patient-centered communication skills) to 55 (representing the highest patient-centered communication skills). Higher scores suggest greater patient-centered communication skills in nurses and vice versa.

### Practical implications

The availability of the Persian version of the mentioned scale as a practical and concise instrument enables the achievement of three goals: (a) assessing patient-centered communication as a unidimensional construct, (b) identifying factors associated with this concept in clinical settings, and (c) determining the effectiveness of different interventions to enhance this particular type of communication.

### Limitations, strengths, and suggestions

Utilizing convenience sampling rather than random sampling approaches, especially stratified random sampling based on the department of practice, not only introduces selection bias, but also limits the generalizability of the findings, as the individuals who enter the study may not necessarily be fully representative of the target population. Conversely, clinical nurses working on the same shift in the same department may also complete the data collection instruments during breaks in the presence of other colleagues. This can, while potentially reducing the participants’ autonomy, lead to biases such as group conformity or social desirability. Because they may respond to questions in a participatory manner and coordinate their answers with the opinions of other colleagues (group conformity bias). They may also provide answers that appear more socially or professionally desirable than what they truly believe (social desirability bias).

Furthermore, although the acceptable approximate normality of the data (as evidenced by skewness and kurtosis within conventional limits) and the five-point ordinal nature of the items (which performs well with ML estimation in sufficient sample sizes) justify the use of this estimator in factor analyses, ML estimation theoretically assumes variables continuous. Therefore, alternative estimators specifically designed for categorical/ordinal data, such as diagonally weighted least squares (DWLS) or robust weighted least squares-mean and variance adjusted (WLSMV), could be considered to further confirm the stability of the factor structure in future cross-validation studies. Additionally, because the present study focused on the translation, cultural adaptation, and evaluation of the psychometric features of the Persian version of the PCCS, it is recommended that future research provide further evidence of the scale’s validity, particularly regarding nomological validity (which tests the scale’s relationships with theoretically related constructs) and criterion validity (which determines the scale’s relationship with appropriate external indicators) to enhance its research and clinical applications.

Moreover, all items of the Persian version of the PCCS are worded in the same positive direction. While this enhances internal consistency and conceptual clarity, it may increase the possibility of straight-line responding, where respondents endorse the same option across all items without careful consideration of each item’s content. Although the observed variability in total scores (range 11–55; SD ≈ 14.8) suggests that uniform responding was not pervasive, this potential bias cannot be entirely ruled out. Therefore, future cross-validation studies are encouraged to incorporate reverse-scored items or employ statistical approaches for detecting careless responses (e.g., long-string analysis, Mahalanobis distance, or response time monitoring) to further strengthen the scale’s validity and reduce the impact of response sets.

While considering these limitations, the present study, by implementing a step-by-step and systematic approach, as well as a strong and precise methodology, provided valuable evidence regarding the validity and reliability of the PCCS in Iranian clinical nurses.

## Conclusions

The Persian version of the PCCS not only exhibited favorable validity through various forms, such as face, content, construct, and convergent validity, but also showed satisfactory reliability through multiple approaches, including internal consistency, construct reliability, and external stability. However, due to the single-center data collection, caution should be exercised in generalizing the results to clinical nurses working in medical centers linked to other Iranian universities of medical sciences and non-university facilities, such as private medical centers.

## Supporting information

S1 Appendix Data collection instruments. (DOCX)

## References

[1] Fite RO, Assefa M, Demissie A, Belachew T. Predictors of therapeutic communication between nurses and hospitalized patients. Heliyon. 2019;5(10):e02665. doi: 10.1016/j.heliyon.2019.e02665 31720457 PMC6838810

[2] Kwame A, Petrucka PM. A literature-based study of patient-centered care and communication in nurse-patient interactions: barriers, facilitators, and the way forward. BMC Nurs. 2021;20(1):158. doi: 10.1186/s12912-021-00684-2 34479560 PMC8414690

[3] Burgener AM. Enhancing communication to improve patient safety and to increase patient satisfaction. Health Care Manag (Frederick). 2017;36(3):238–43. doi: 10.1097/HCM.0000000000000165 28657914

[4] Khoir M. Therapeutic communication skills of nurses in hospital. Int J Nurs Health Serv. 2020;3(2):275–83.

[5] Kim B, Lee SY, An GJ, Lee G, Yun HJ. Influence of communication competency and nursing work environment on job satisfaction in hospital nurses. J Health Info Stat. 2019;44(2):189–97. doi: 10.21032/jhis.2019.44.2.189

[6] Kim NR, Kim SE, Jang SE. The effects of communication ability, job satisfaction, and organizational commitment on nursing performance of intensive care unit nurses. J Korean Crit Care Nurs. 2022;15(1):58–68. doi: 10.34250/jkccn.2022.15.1.58

[7] Boeykens D, Boeckxstaens P, De Sutter A, Lahousse L, Pype P, De Vriendt P, et al. Goal-oriented care for patients with chronic conditions or multimorbidity in primary care: A scoping review and concept analysis. PLoS One. 2022;17(2):e0262843. doi: 10.1371/journal.pone.0262843 35120137 PMC8815876

[8] McCabe C. Nurse-patient communication: an exploration of patients’ experiences. J Clin Nurs. 2004;13(1):41–9. doi: 10.1111/j.1365-2702.2004.00817.x 14687292

[9] Joo Y, Jang Y, Park CG, Yang YL. Development and validation of a patient-centered communication scale for nurses. BMC Nurs. 2024;23(1):550. doi: 10.1186/s12912-024-02174-7 39135182 PMC11320938

[10] Slatore CG, Hansen L, Ganzini L, Press N, Osborne ML, Chesnutt MS, et al. Communication by nurses in the intensive care unit: qualitative analysis of domains of patient-centered care. Am J Crit Care. 2012;21(6):410–8. doi: 10.4037/ajcc2012124 23117904 PMC3992836

[11] Mersha A, Abera A, Tesfaye T, Abera T, Belay A, Melaku T, et al. Therapeutic communication and its associated factors among nurses working in public hospitals of Gamo zone, southern Ethiopia: application of Hildegard Peplau’s nursing theory of interpersonal relations. BMC Nurs. 2023;22(1):381. doi: 10.1186/s12912-023-01526-z 37833693 PMC10571273

[12] Wune G, Ayalew Y, Hailu A, Gebretensaye T. Nurses to patients communication and barriers perceived by nurses at Tikur Anbessa Specialized Hospital, Addis Ababa, Ethiopia. Int J Afr Nurs Sci. 2020;12:100197. doi: 10.1016/j.ijans.2020.100197

[13] LS L. Strategies for effective patient communication. 2018. Available from: https://www.americannursetoday.com/blog/strategies-for-effective-patient-communication/

[14] Hur G. Construction and validation of a global interpersonal communication competence scale. Korean J Commun Stud. 2003;47(6):380.

[15] Alshammari M, Duff J, Guilhermino M. Psychometric evaluation of the Arabic version of the patient-centered communication instrument for adult cancer patients. Int J Qual Health Care. 2021;33(1):mzaa159. doi: 10.1093/intqhc/mzaa159 33274737

[16] Demiris G, Pitzer K, Washington K, Oliver DP. Reliability and validity testing of the caregiver-centered communication questionnaire. J Nurs Meas. 2023;31(3):439–47. doi: 10.1891/JNM-2022-0044 35680422 PMC9732140

[17] Amin SM, Abdallah HMM, Hamad NIM, Wanas AHB, Elhapashy HMM, Yehia AYM, et al. The moral middle ground: How moral distress mediates the link between patient-centered communication and palliative caring competence among oncology nurses. Eur J Oncol Nurs. 2025;79:103003. doi: 10.1016/j.ejon.2025.103003 41092521

[18] Jalali A, Sharifi A, Babaei K, Moradi K, Karimi J, Moradi L, et al. Psychometric properties of the Persian version of the Nursing Work Environment Scale during public health emergencies. Crit Public Health. 2026;36(1). doi: 10.1080/09581596.2026.2683748

[19] Jafari Rahim Abad Y, Saadati K, Ghaffari F. Psychometric evaluation of the ethical caring competency scale among Iranian Nurses: A methodological study. PLoS One. 2026;21(5):e0346672. doi: 10.1371/journal.pone.0346672 42102056 PMC13155570

[20] Tehranineshat B, Rivaz M, Kargar Dolatabadi E. Psychometric testing of the Persian version of the Nursing Care Quality Scale: A methodological study. Nurs Open. 2023;10(9):6491–500. doi: 10.1002/nop2.1900 37322844 PMC10416060

[21] Wild D, Grove A, Martin M, Eremenco S, McElroy S, Verjee-Lorenz A, et al. Principles of Good Practice for the Translation and Cultural Adaptation Process for Patient-Reported Outcomes (PRO) Measures: report of the ISPOR Task Force for Translation and Cultural Adaptation. Value Health. 2005;8(2):94–104. doi: 10.1111/j.1524-4733.2005.04054.x 15804318

[22] Jalali A, Naghibzadeh A, Mohammadi MM, Rostami M, Kalhory P, Taghvostani NM, et al. Translation and validation of the Persian version of the Nursing Practice Readiness Scale (NPRS) for new graduate nurses. BMC Nurs. 2024;23(1):760. doi: 10.1186/s12912-024-02434-6 39415162 PMC11484381

[23] Jalali A, Darvishi N, Kalhory P, Merati F, Vatandost S, Moradi K. Intensive care unit dignified care: Persian translation and psychometric evaluation. Nurs Open. 2024;11(7):e2238. doi: 10.1002/nop2.2238 38978289 PMC11231042

[24] Hoseinzadeh E, Sharif-Nia H, Ashktorab T, Ebadi A. Development and psychometric evaluation of nurse’s intention to care for patients with infectious disease scale: an exploratory sequential mixed method study. BMC Nurs. 2024;23(1):65. doi: 10.1186/s12912-023-01669-z 38267951 PMC10807223

[25] Shahidi Delshad E, Soleimani M, Zareiyan A, Ghods AA. Development and psychometric properties evaluation of nurses’ innovative behaviours inventory in Iran: protocol for a sequential exploratory mixed-method study. BMJ Open. 2024;14(2):e077056. doi: 10.1136/bmjopen-2023-077056 38316597 PMC10860078

[26] Bahrami N, Yaghoobzadeh A, Sharif Nia H, Soliemani MA, Haghdoost AA. Psychometric properties of the Persian version of Larsons sexual satisfaction questionnaire in a sample of Iranian infertile couples. Iran J Epidemiol. 2016;12(2):18–31.

[27] Sharif-Nia H, Alikari V, Marôco J, Fatehi R, Hoseinzadeh E, Nowrozi P. Psychometric properties of the Greek simplified medication adherence questionnaire among Iranian hemodialysis patients. Sci Rep. 2024;14(1):28372. doi: 10.1038/s41598-024-80134-6 39551814 PMC11570638

[28] Kaveh O, Sharif-Nia H, Hosseini Z, Kaur H, Shafipour V. Psychometrics evaluation of the Persian version of Attitudes toward Patient Safety Questionnaire (APSQ-III) in nursing students. BMC Med Educ. 2024;24(1):1419. doi: 10.1186/s12909-024-06318-w 39633360 PMC11619130

[29] Lawshe C. A quantitative approach to content validity. Pers Psychol. 1975. doi: 10.1111/j.1744-6570.1975.tb01393.x

[30] EghbalManesh A, Ebadi A, Zoladl M, Rasoul Zadeh Haghighi Z. Psychometric properties of the Persian version of the electroconvulsive related anxiety questionnaire for psychiatric patients. Front Psychol. 2025;16:1472259. doi: 10.3389/fpsyg.2025.1472259 40040656 PMC11876117

[31] Polit D, Beck C. Essentials of nursing research: appraising evidence for nursing practice. Lippincott Williams & Wilkins; 2020.

[32] Roebianto A, Savitri SI, Aulia I, Suciyana A, Mubarokah L. Content validity: Definition and procedure of content validation in psychological research. TPM Test Psychom Methodol Appl Psychol. 2023;30(1):5–18. doi: 10.4473/TPM30.1.1

[33] Polit-O’Hara D, Yang FM. Measurement and the measurement of change: a primer for the health professions. 2016.

[34] Hosseini L, Sharif Nia H, Ashghali Farahani M. Development and Psychometric Evaluation of Family Caregivers’ Hardiness Scale: A Sequential-Exploratory Mixed-Method Study. Front Psychol. 2022;13:807049. doi: 10.3389/fpsyg.2022.807049 35432109 PMC9010881

[35] Sharif-Nia H, Marôco J, Hoseinzadeh E, Moshtagh M, Hatamipour K. Validity and reliability of the Persian version of the gender equity scale in nursing education. BMC Nurs. 2025;24(1):187. doi: 10.1186/s12912-025-02831-5 39966868 PMC11837590

[36] Sharif-Nia H, She L, Osborne J, Gorgulu O, Khoshnavay Fomani F, Goudarzian AH. Statistical concerns, invalid construct validity, and future recommendations. Nurs Pract Today. 2024;11(1). doi: 10.18502/npt.v11i1.14938

[37] Ghasempour S, Bagheri H, Sharif-Nia H, Marôco J, Miraghai F, Abbasi A. Psychometric characteristics of the Persian version of the State Self-Compassion Scale in patients with cardiovascular diseases. Acta Psychol (Amst). 2026;266:106942. doi: 10.1016/j.actpsy.2026.106942 42061068

[38] Fornell C, Larcker DF. Evaluating structural equation models with unobservable variables and measurement error. J Mark Res. 1981;18(1):39–50. doi: 10.1177/002224378101800104

[39] Sharif Nia H, Pahlevan Sharif S, Boyle C, Yaghoobzadeh A, Tahmasbi B, Rassool GH, et al. The Factor structure of the spiritual well-being scale in veterans experienced chemical weapon exposure. J Relig Health. 2018;57(2):596–608. doi: 10.1007/s10943-017-0458-1 28748326

[40] Allen MJ, Yen WM. Introduction to Measurement Theory. Brooks/Cole Publishing Company; 1979.

[41] Marx RG, Menezes A, Horovitz L, Jones EC, Warren RF. A comparison of two time intervals for test-retest reliability of health status instruments. J Clin Epidemiol. 2003;56(8):730–5. doi: 10.1016/s0895-4356(03)00084-2 12954464

[42] Hosseini FA, Momennasab M, Guàrdia-Olmos J, Yektatalab S, Shaygan M, Zareiyan A. Designing and psychometric properties of the hospitalized patients’ spiritual needs questionnaire (HPSNQ) in the medical-surgical hospital setting. BMC Palliat Care. 2023;22(1):112. doi: 10.1186/s12904-023-01213-5 37542263 PMC10403866

[43] Epstein RM, Street RL Jr. The values and value of patient-centered care. Ann Fam Med. 2011;9(2):100–3. doi: 10.1370/afm.1239 21403134 PMC3056855

[44] Street RL Jr, Makoul G, Arora NK, Epstein RM. How does communication heal? Pathways linking clinician-patient communication to health outcomes. Patient Educ Couns. 2009;74(3):295–301. doi: 10.1016/j.pec.2008.11.015 19150199

[45] Sousa VD, Rojjanasrirat W. Translation, adaptation and validation of instruments or scales for use in cross-cultural health care research: a clear and user-friendly guideline. J Eval Clin Pract. 2011;17(2):268–74. doi: 10.1111/j.1365-2753.2010.01434.x 20874835

[46] Pahlevan Sharif S, Bolt EET, Ahadzadeh AS, Turner JJ, Sharif Nia H. Organisational support and turnover intentions: A moderated mediation approach. Nurs Open. 2021;8(6):3606–15. doi: 10.1002/nop2.911 33979031 PMC8510732

[47] Watkins MW. Exploratory factor analysis: A guide to best practice. J Black Psychol. 2018;44(3):219–46. doi: 10.1177/0095798418771807

[48] Brown TA. Confirmatory factor analysis for applied research. Guilford Publications; 2015.

[49] Zhang Z, Yuan K-H. Robust coefficients alpha and omega and confidence intervals with outlying observations and missing data: methods and software. Educ Psychol Meas. 2016;76(3):387–411. doi: 10.1177/0013164415594658 29795870 PMC5965561

[50] Trizano-Hermosilla I, Alvarado JM. Best alternatives to Cronbach’s alpha reliability in realistic conditions: Congeneric and asymmetrical measurements. Front Psychol. 2016;7:769. doi: 10.3389/fpsyg.2016.00769 27303333 PMC4880791

[51] Swan K, Speyer R, Scharitzer M, Farneti D, Brown T, Woisard V, et al. Measuring what matters in healthcare: a practical guide to psychometric principles and instrument development. Front Psychol. 2023;14:1225850. doi: 10.3389/fpsyg.2023.1225850 37790221 PMC10543275

