# Supplementary material for: Psychometric features of the Patient-Centered Communication Scale among Iranian clinical nurses
Source: PLoS One. 2026 Sep 18;21(9):e0358575. doi: 10.1371/journal.pone.0358575 (PMC13588382; doi:10.1371/journal.pone.0358575)
Supplement: Supplementary file 2 [file pone.0358575.s004.pdf]

Editor: PLOS One.

August 27, 2026

Dear Dr Maria José Nogueira;

Thank you very much for considering our revised manuscript, "**Psychometric features of the Patient-Centered Communication Scale among Iranian clinical nurses**". Manuscript Number: **PONE-D-26-05593** for potential placement in PLOS One.

We have revised the manuscript in accordance with the editorial recommendations. Our responses are given in a point-by-point manner in the grid that is presented below. In the revised manuscript, we have highlighted the corrected text in accordance with the reviewers' and editor's recommendations.

We sincerely appreciate the opportunity to provide these revisions to the manuscript and believe the recommendations have continued to improve the paper. We hope the revised version is now suitable for publication and look forward to hearing from you in this regard.

Sincerely,

Reviewers Comments	***Author response***
Journal Requirements	
If the reviewer comments include a recommendation to cite specific previously published works, please review and evaluate these publications to determine whether they are relevant and should be cited. There is no requirement to cite these works unless the editor has indicated otherwise.	Thank you for your attention to detail.<br>The reviewers did not recommend any articles for citation.
Please review your reference list to ensure that it is complete and correct. If you have cited papers that have been retracted, please include the rationale for doing so in the manuscript text, or remove these references and replace them with relevant current references. Any changes to the reference list should be mentioned in the rebuttal letter that accompanies your revised manuscript. If you need to cite a retracted article, indicate the article's retracted status in the References list and also include a citation and full reference for the retraction notice.	The list of references was re-examined.<br>While confirming its correctness and completeness, this manuscript does not cite any retracted articles.
Additional Editor Comments	
The authors have revised the manuscript in accordance with the recommendations from the previous review round, and the overall quality of the manuscript has improved substantially.<br>However, several issues identified during the initial review remain insufficiently addressed and require further clarification before the manuscript can be considered for acceptance.<br>The authors are therefore requested to carefully address the additional comments provided by the reviewers and revise the manuscript accordingly.	Thank you for your encouraging words.<br>The authors sincerely appreciate the esteemed reviewers and the respected editor for their positive evaluation of the revised manuscript.<br>We also tried to carefully review all the ambiguities raised and make the necessary corrections in the current version in accordance with the reviewers' suggestions.
Reviewer #1	
The authors have responded to comments from myself and the other reviewers thoroughly and I believe the manuscript can get accepted with very minor edits.	Thank you for your encouraging words.<br>It is hoped that the following amendments will enhance the quality of this manuscript.
One suggestion would be to split the data presented in Table 1 so that the two independently drawn samples of 150 nurses can be easily compared rather than collapsed across. They could even be formally compared via stats.	Demographic characteristics are presented separately for the first and second independent samples in Table 1.<br>These samples were also formally compared on

	these variables using statistical tests.<br>In addition, to maintain the sequence of content in both the Materials and methods and the Results, the "Demographic characteristics of participants" section was moved before the "Construct validity" section.<br>The changes have been highlighted in yellow.
Second, Table 3 shows that the unidimensional model without correlating residuals provided a substantially worse fit than the three-factor model. But then only the unidimensional model was afforded the correlated residuals. I expect nothing would change as the unidimensional solution would remain more parsimonious - but the same affordance should be made for the three-factor model (of course if it was the case that MI-s did not exceed the threshold the authors identified then this is fine).	We sincerely thank the reviewer for this insightful methodological observation.<br>You are absolutely correct in noting the asymmetry in the use of correlated residuals between the unidimensional and three-factor models.<br>To clarify: we initially tested the three-factor model on the second independent sample (n=150) and obtained a satisfactory fit ($\chi^2(39)=104.52$, $P<0.001$, $\chi^2/df=2.68$, RMSEA=0.08, CFI=0.98, TLI=0.97, PNFI=0.72, and PCFI=0.73). Because this model already met the conventional fit thresholds, we did not inspect its modification indices for residual covariances, as further adjustments were not required to establish adequate fit.<br>In contrast, the unidimensional model (without correlated residuals) showed a suboptimal fit ($\chi^2(44)=185.24$, $P<0.001$, $\chi^2/df=4.21$, RMSEA=0.12, CFI=0.89, TLI=0.87, PNFI=0.47, and PCFI=0.48). To improve this

	model, we followed standard CFA practice and examined modification indices, adding only two theoretically justified residual covariances (items 11-12 and items 6-7). This brought the unidimensional model to an excellent fit.<br>The critical comparison between the two structures, however, was based on the final models, the three-factor model (which already fit well without any residual covariances) and the revised unidimensional model. The chi-square difference test ($\Delta\chi^2(3)=4.21$, $P=0.24$) was not significant, and the parsimony indices (AIC, BIC) clearly favored the more parsimonious unidimensional structure. Thus, even if we had examined and added residual covariances to the three-factor model (which was unnecessary given its already acceptable fit), the outcome of the model comparison would remain unchanged: the unidimensional model is more parsimonious and statistically equivalent in fit.
Third, now that I've revisited the scale, it occurs to me that all of the items are in the same direction and so straight-line responding is a possibility. It is also difficult to control for or identify as it would not be unreasonable to expect the same response on all items.	We sincerely thank the reviewer for this insightful methodological observation.<br>We fully agree that all items of the Persian version of the PCCS are worded in the same positive direction, which indeed raises the possibility of straight line responding (i.e., respondents uniformly selecting the same Likert option without careful discrimination).

	We acknowledge that this is a common limitation in unidimensional, positively framed self report instruments, and it is particularly difficult to detect or control statistically in cross sectional designs. However, we would like to note two points: (a) our sample demonstrated adequate variability in total scores (range 11–55, SD≈14.8), suggesting that not all participants engaged in careless uniform responding; and (b) the excellent model fit and high factor loadings (0.77–0.96) indicate that the items strongly cohere around the latent construct, which somewhat mitigates concerns about random responding.<br>To address this issue transparently, we have added a brief statement to the Limitations, strengths, and suggestions subsection of the Discussion, acknowledging this potential bias and recommending that future studies consider including reverse scored items or employing careless response detection methods (e.g., longstring analysis, Mahalanobis distance, or response time monitoring) to further strengthen the scale's validity.<br>We are grateful for this valuable comment, which has improved the methodological rigor of our manuscript.
Fourth, it occurs to me that the mean, median, SD, range and other descriptive parameters for the total scale score were not reported anywhere. I'd suggest doing so for both of the samples only for the	Thank you for your valuable comment.<br>Descriptive indices such as mean, median,

unidimensional solution. This is useful data to people considering the scale.	standard deviation (SD), and range (minimum and maximum) for the overall scores of the single-factor structure of this scale were calculated separately for the first and second independent samples.<br>The changes have been highlighted in yellow.
Finally, in the discussion - is there a reason to assume that relationship status would / should impact this scale? If not, I'd drop that bit. The big difference in work experience however is a good addition.	Thank you for your constructive feedback.<br>The sentences regarding the apparent difference between the marital status of the participants in the current study and the initial study of scale design were removed from the "Discussion", and only the apparent difference between their work experience was emphasized as one of the possible reasons for the difference in the dimensionality of the original and Persian versions of the Patient-Centered Communication Scale.<br>The changes have been highlighted in yellow.
Reviewer #2	
[1] Unifactorial or tri-factorial?<br>Comment: Ok	Thank you very much.
[2] Viewpoint of nurses<br>Comment: Ok	Thank you very much.
[3] normal X "robust to moderate violations of normality"<br>>> 1st submission - p.13 and 15) "indicated normal univariate and multivariate distribution of the items, respectively."<br>>> R1 - p.16) "Skewness values spanning -3.00 to +3.00 and kurtosis values spanning -7.00 to +7.00, as well as Mardia coefficient values less than 8.00, indicated normal univariate and multivariate distribution of the items, respectively."	Thank you for your attention to detail.<br>The phrase "indicated normal univariate and multivariate distribution of the items" was changed to "indicated no substantial deviation from the univariate and multivariate normal distribution of the items".

Comment: OK, "ML estimation is robust to moderate violations of normality"<br>But we cannot conclude that these items have a normal distribution.<br>Suggestion: Remove this sentence or cite methodological authors that justify this statement.	The changes have been highlighted in yellow.<br>In addition, skewness values spanning -3.00 to +3.00 and kurtosis values spanning -7.00 to +7.00, as well as Mardia coefficient values less than 8.00, were also reported based on the following references in the "Normal distribution and outlier values" section of the Materials and methods.<br>• *Sharif-Nia H, Marôco J, Hoseinzadeh E, Moshtagh M, Hatamipour K. Validity and reliability of the Persian version of the gender equity scale in nursing education. BMC Nurs. 2025;24(1):187. doi: 10.1186/s12912-025-02831-5.*<br>• *Ghasempour S, Bagheri H, Sharif-Nia H, Marôco J, Miraghai F, Abbasi A. Psychometric characteristics of the Persian version of the State Self-Compassion Scale in patients with cardiovascular diseases. Acta Psychol. 2026;266:106942. doi: 10.1016/j.actpsy.2026.106942.*
[4] Correlations between the error terms<br>Comment: Ok	Thank you very much.
[5] Persian version as supplementary files.<br>Comment: Ok	Thank you very much.
