# Supplementary material for: Psychometric features of the Patient-Centered Communication Scale among Iranian clinical nurses
Source: PLoS One. 2026 Sep 18;21(9):e0358575. doi: 10.1371/journal.pone.0358575 (PMC13588382; doi:10.1371/journal.pone.0358575)
Supplement: Supplementary file 3 [file pone.0358575.s001.docx]

**چک‌لیست ویژگی‌های دموگرافیک**

**با سلام و احترام**

**همکار عزیز و ارجمند**،

این مطالعه با هدف **ترجمه و روان‌سنجی مقیاس ارتباط بیمارمحور (PCCS) در پرستاران بالینی ایرانی** آماده شده است.

لازم به ذکر است پاسخ‌های شما کاملاً محرمانه خواهد بود و نیازی به ذکر اطلاعات هویتی و نام و نام خانوادگی نیست.

پیشاپیش از همکاری شما در پاسخ‌گویی صادقانه بی‌نهایت سپاس‌گزاریم.

**با تشکر و سپاس فراوان**

**سن (به سال ذکر شود.):** ....................

**جنسیت:** زن □ / مرد □

**وضعیت تاهل:** متاهل □ / مجرد □ / مطلقه □ / بیوه □

**سطح تحصیلات:** کارشناسی پرستاری □ / کارشناسی‌ارشد پرستاری □

**سابقه کاری (به سال ذکر شود.):** ....................

**بخش محل فعالیت:** شیمی‌درمانی □ / داخلی □ / گوارش □ / قلب □ / پس از مراقبت‌های ویژه قلب □ / بیماری‌های عفونی □ / جراحی □ / ارولوژی □ / ارتوپدی □ / دیالیز □ / مراقبت‌های ویژه □ / مراقبت‌های ویژه قلب □ / اورژانس □ / اطفال و نوزادان □ / سایر □

**وضعیت استخدامی:** رسمی □ / غیررسمی □

**کفایت درآمد:** تا چه اندازه درآمد ماهیانه خود را برای هزینه‌های زندگی کافی می‌دانید؟

پایین‌تر از حد کفاف □ / در حد کفاف □ / بالاتر از حد کفاف □

**نسخه فارسی مقیاس ارتباط بیمارمحور**

پاسخ‌دهنده گرامی،

لطفاً میزان موافقت خود را با هر یک از جملات زیر مشخص نمایید.

| **ردیف** | **عبارت** | **کاملاً موافقم** | **تا حدی موافقم** | **نه موافقم و نه مخالفم** | **تا حدی مخالفم** | **کاملاً مخالفم** |
| --- | --- | --- | --- | --- | --- | --- |
| 1 | **هنگام برقراری ارتباط، فعالانه به سخنان بیمار یا خانواده‌اش گوش می‌دهید.** |  |  |  |  |  |
| 2 | **بخش‌ها یا سازمان‌های دیگری که می‌توانند به بیمار یا خانواده‌اش کمک کنند (به‌عنوان مثال، تیم مددکاری اجتماعی، انجمن مراقبان، روان‌درمانی و غیره) را معرفی می‌کنید.** |  |  |  |  |  |
| 3 | **دلیل ضرورت معالجه پزشکی، روند درمانی یا مداخله پرستاری را برای بیمار یا خانواده‌اش شرح می‌دهید.** |  |  |  |  |  |
| 4 | **جهت ایجاد مداخلات درمانی و کمک به درک بیمار یا خانواده‌اش، با رشته‌های مختلف (به‌عنوان مثال، پزشکان، متخصص تغذیه، فیزیوتراپ و غیره) همکاری می‌کنید.** |  |  |  |  |  |
| 5 | **زمان کافی برای پرسیدن سؤالات را در اختیار بیمار یا خانواده‌اش قرار می‌دهید.** |  |  |  |  |  |
| 6 | **اطلاعاتی که بیمار یا خانواده‌اش می‌خواهند بدانند را شناسایی کرده و به موقع ارائه می‌دهید.** |  |  |  |  |  |
| 7 | **از دیدگاه بیمار یا خانواده‌اش به موقعیت نگاه می‌کنید و با آن‌ها ارتباط برقرار می‌کنید.** |  |  |  |  |  |
| 8 | **پس از ارائه اطلاعات، سطح درک بیمار یا خانواده‌اش را بررسی می‌کنید.** |  |  |  |  |  |
| 9 | **بیمار یا خانواده‌اش را تشویق می‌کنید تا هرگونه احساسی را که ممکن است در رابطه با بیماری تجربه نمایند، بیان کنند.** |  |  |  |  |  |
| 10 | **در حین ارائه مراقبت، با همکاران خود و یا کادر درمان (به‌عنوان مثال، پزشک، همکار پرستار و غیره) ارتباط برقرار می‌کنید و احساسات منفی و مثبت خود درباره بیمار یا خانواده‌اش را با آن‌ها به اشتراک می‌گذارید.** |  |  |  |  |  |
| 11 | برای بیان آزادانه **احساسات، نیازها و نظرات** **بیمار یا خانواده‌اش** **از سؤالات باز استفاده می‌کنید. (مثلاً ”امروز چه احساسی دارید؟“)** |  |  |  |  |  |

**Demographic characteristics checklist**

**Greetings and regards.**

**Dear and esteemed colleague,**

This study was prepared with the aim of **translating and psychometrically evaluating the Patient-Centered Communication Scale (PCCS) in Iranian clinical nurses**.

It should be noted that your responses will be completely confidential, and there is no need to mention your identity information, name, and surname.

We thank you in advance for your cooperation in answering honestly.

**With many thanks and gratitude**

**Age (in years):** ....................

**Gender:** Male □ / Female □

**Marital status:** Married □ / Single □ / Divorced □ / Widow □

**Education level:** Bachelor of Science in Nursing □ / Master of Science in Nursing □

**Work experience (in years):** ....................

**Department of practice:** Chemotherapy □ / Internal medicine □ / Gastroenterology □ / Cardiology □ / Post cardiac care unit □ / Infectious diseases □ / Surgery □ / Urology □ / Orthopedics □ / Emergency rooms □ / Pediatrics and neonatology □ / Others □

**Employment status:** Officially □ / Non-officially □

**Income adequacy:** To what extent do you consider your monthly income adequate for living expenses?

Below average □ / Average □ / Above average □

**Persian version of the Patient-Centered Communication Scale**

Dear Respondent,

Please indicate your level of agreement with each of the following statements.

| **No.** | **Item** | **Strongly agree** | **Somewhat agree** | **Neutral** | **Somewhat disagree** | **Strongly disagree** |
| --- | --- | --- | --- | --- | --- | --- |
| **1** | Listen actively to the words of the patient and family members when communicating. |  |  |  |  |  |
| **2** | Introduce departments or other organizations that can help the patient or family (e.g., social work team, caregiver association, psychotherapy etc.) |  |  |  |  |  |
| **3** | Describe the reason for the necessity of medical treatment/procedures or nursing intervention to the patient or their family. |  |  |  |  |  |
| **4** | Collaborate across disciplines (e.g., physicians, nutritionist, physical therapist, etc.) to establish therapeutic interventions and aid patient and/or family's understanding. |  |  |  |  |  |
| **5** | Provide enough time to ask questions. |  |  |  |  |  |
| **6** | Identify the information patient/families want to know and provide it in a timely manner. |  |  |  |  |  |
| **7** | Look at the situation from the patient or family's point of view and communicate with them. |  |  |  |  |  |
| **8** | Check the patient or family's level of understanding after providing the information. |  |  |  |  |  |
| **9** | Encourage the patient or family to express any feelings they may experience related to the illness. |  |  |  |  |  |
| **10** | Communicate and share my negative and positive emotions about the patient or their family while providing care with my fellows and/or medical staff (e.g., physician, fellow nurse, etc.) |  |  |  |  |  |
| **11** | Use open-ended questions to allow the patient and family to express their feelings, needs, and opinions freely (e.g., "How are you feeling today?") |  |  |  |  |  |
