# Supplementary material for: Psychometric features of the Patient-Centered Communication Scale among Iranian clinical nurses
Source: PLoS One. 2026 Sep 18;21(9):e0358575. doi: 10.1371/journal.pone.0358575 (PMC13588382; doi:10.1371/journal.pone.0358575)
Supplement: Peer review history

# Peer review: Decision Letter 0

**DOI:** 10.1371/journal.pone.0358575.r001

**Authors:** Maria José Nogueira

24 Apr 2026

Dear Dr. Ghasempour,

Thank you for submitting your manuscript to PLOS ONE. After careful consideration, we feel that it has merit but does not fully meet PLOS ONE’s publication criteria as it currently stands. Therefore, we invite you to submit a revised version of the manuscript that addresses the points raised during the review process.

Please submit your revised manuscript by Jun 08 2026 11:59PM. If you will need more time than this to complete your revisions, please reply to this message or contact the journal office at plosone@plos.org. When you're ready to submit your revision, log on to https://www.editorialmanager.com/pone/ and select the 'Submissions Needing Revision' folder to locate your manuscript file.

- A letter that responds to each point raised by the academic editor and reviewer(s). You should upload this letter as a separate file labeled 'Response to Reviewers'.
- A marked-up copy of your manuscript that highlights changes made to the original version. You should upload this as a separate file labeled 'Revised Manuscript with Track Changes'.
- An unmarked version of your revised paper without tracked changes. You should upload this as a separate file labeled 'Manuscript'.

If you would like to make changes to your financial disclosure, please include your updated statement in your cover letter. Guidelines for resubmitting your figure files are available below the reviewer comments at the end of this letter.

If applicable, we recommend that you deposit your laboratory protocols in protocols.io to enhance the reproducibility of your results. Protocols.io assigns your protocol its own identifier (DOI) so that it can be cited independently in the future. For instructions see: https://journals.plos.org/plosone/s/submission-guidelines#loc-laboratory-protocols. Additionally, PLOS ONE offers an option for publishing peer-reviewed Lab Protocol articles, which describe protocols hosted on protocols.io. Read more information on sharing protocols at https://plos.org/protocols?utm_medium=editorial-email&utm_source=authorletters&utm_campaign=protocols.

As the corresponding author, your ORCID iD is verified in the submission system and will appear in the published article. PLOS supports the use of ORCID, and we encourage all coauthors to register for an ORCID iD and use it as well. Please encourage your coauthors to verify their ORCID iD within the submission system before final acceptance, as unverified ORCID iDs will not appear in the published article. *Only*  the individual author can complete the verification step; PLOS staff *cannot*  verify ORCID iDs on behalf of authors.

We look forward to receiving your revised manuscript.

Kind regards,

Maria José Nogueira, Ph.D.

Academic Editor

PLOS One

**Journal Requirements:**

When submitting your revision, we need you to address these additional requirements.

1. Please ensure that your manuscript meets PLOS ONE's style requirements, including those for file naming. The PLOS ONE style templates can be found at

https://journals.plos.org/plosone/s/file?id=wjVg/PLOSOne_formatting_sample_main_body.pdf and

https://journals.plos.org/plosone/s/file?id=ba62/PLOSOne_formatting_sample_title_authors_affiliations.pdf

2. In the online submission form you indicate that your data is not available for proprietary reasons and have provided a contact point for accessing this data. Please note that your current contact point is a co-author on this manuscript. According to our Data Policy, the contact point must not be an author on the manuscript and must be an institutional contact, ideally not an individual. Please revise your data statement to a non-author institutional point of contact, such as a data access or ethics committee, and send this to us via return email. Please also include contact information for the third party organization, and please include the full citation of where the data can be found.

3. You have indicated that data is available from [vcr@shmu.ac.ir].  Please can we ask you to provide us with a general contact email address for the data requests, so readers can request access in perpetuity. If a general email is not available please provide a link to a website where readers can obtain access to data.

4. Please upload a new copy of Figure 1 as the detail is not clear. Please follow the link for more information:  https://journals.plos.org/plosone/s/figures

5. If the reviewer comments include a recommendation to cite specific previously published works, please review and evaluate these publications to determine whether they are relevant and should be cited. There is no requirement to cite these works unless the editor has indicated otherwise.

6. Please review your reference list to ensure that it is complete and correct. If you have cited papers that have been retracted, please include the rationale for doing so in the manuscript text, or remove these references and replace them with relevant current references. Any changes to the reference list should be mentioned in the rebuttal letter that accompanies your revised manuscript. If you need to cite a retracted article, indicate the article’s retracted status in the References list and also include a citation and full reference for the retraction notice.

**Additional Editor Comments:**

Dear authors,

The manuscript is very interesting and highly relevant to the field of nursing.

reviewers have provided important and valuable suggestions.

Please consider all comments carefully and adress all recommendations made by all three reviewers.

[Note: HTML markup is below. Please do not edit.]

Reviewers' comments:

Reviewer's Responses to Questions

**Comments to the Author**

1. Is the manuscript technically sound, and do the data support the conclusions?

Reviewer #1: Yes

Reviewer #2: Yes

Reviewer #3: Yes

**********

2. Has the statistical analysis been performed appropriately and rigorously?

Reviewer #1: Yes

Reviewer #2: No

Reviewer #3: Yes

**********

3. Have the authors made all data underlying the findings in their manuscript fully available?

The PLOS Data policy

Reviewer #1: No

Reviewer #2: No

Reviewer #3: No

**********

4. Is the manuscript presented in an intelligible fashion and written in standard English?

Reviewer #1: Yes

Reviewer #2: Yes

Reviewer #3: Yes

**********

Reviewer #1: The authors set out to translate the original PCC scale from Joo et al. (2024) to Persian and determine validity/reliability on an Iranian sample of nurses.

I found the paper very well written with a good level of detail but I also have a few fairly major issues with the approach.

The biggest issue is removing item 4 from the original paper (actually item 1 as numbered in Table 2 of the original paper (though it does appear 4th from top in that table). The authors describe a detailed procedure of iterative translation, evaluation and modification of the scale. I fear that the good intention of being thorough has resulted in issues due to too much "pre-processing".

Namely, the original (well, at least the original translation from Korean to English) versions of the two items deemed redundant by the authors are as follows:

Item 1: "Describe the medical treatment/procedure or nursing intervention.”

Item 2. “Describe the reason for the necessity of medical treatment/procedures or nursing intervention.”

The first is about WHAT is being done and the second WHY it is being done. The distinction is quite clear, and although I am not fluent in Persian, I believe it should have been straightforward to translate as such, especially considering the detailed procedure of translating the original.

This indicates that the lengthy pre-processing stage itself, at least in part, homogenized the items. If it made these two items so similar that one was deemed redundant then it may have made the rest more similar and therefore contributed to the strong unidimensional structure when compared to the 3-factor solution of the original.

I am also concerned that such a detailed procedure could not result in this simple distinction between WHAT and WHY as quite a simple one.

I have two further approach-related issues. First, the confirmatory analysis should have compared the unidimensional model to a 3-factor model as proposed by the original paper. From what I can see, it is almost a certainty that absolutely nothing will change and that a unidimensional structure fits better. However, I would still advise doing it as the original paper provides one hypothesis and the first wave of data collection with the adopted version another - these can be neatly compared. And second, I strongly disagree with data not being made available. This is not patient data, and it can be sufficiently anonymized. I would have considered uploading the questionnaire data alongside perhaps age, sex and total work experience.

Considering that I believe that the process by which the items were translated and modified has contributed to the homogeneity of the factor structure - the recommendation is trickier than it might seem. On one hand, the paper is very well written, and the authors have obviously been thorough. Additionally, the scale itself is likely to be useful for researchers from Iran and beyond. On the other hand, the big issue being the only part of the process that cannot be addressed (the pre data collection stage) makes it problematic.

I am suggesting major revisions, if the authors make changes to, primarily, how they discuss differences between the original and their own version (see comments below). I believe this can be published, with some very obvious caveats.

See specific issues for each section below.

Introduction

Lines 84-92 – authors give the argument that the scales usually being used were not designed with nurses in mind – they then mention that others have explored validity and psychometric properties of other scales – but not what the findings were and how that informs their decision. Perhaps there's a bit of a language barrier here as in the examples were meant to enumerate that work has been done but not from nurses' perspective and nothing more. As currently written, it feels like there is more to the argument, something about the properties of these scales which is not mentioned.

Line 95 – should not have the year in parentheses as it isn’t consistent with the referencing style used here.

Methods

Line 115-117 – it is not clear why an undergraduate degree and a year of experience was required for taking part in the study – surely that reduces the possible range of results compared to how it the scale may be used.

Line 264-265 – how were the nurses for test-retest selected? Was this random? If not, it could have biased the estimate.

Results

Table 1 – being too pedantic here – but I would stick to two decimal spots for the % to keep in line with the rest of the paper (even if the value is something like 26.70).

Figure 1 shows that residuals of Item 11&12 as well as 6&7 were allowed to correlate. This was not mentioned in text, what was the criteria by which this was decided? Were modification indices looked at diagnostically then a decision made based on something like method effects due to similar wording? I do think, looking at the content, that the decision is fine - it should just not be glossed over. I would report model indices with and without the modification (perhaps the initial model fit can be a table in an appendix / supplement to keep the main text flow).

Discussion

Considering the issues I mention above, I believe the discussion should be more nuanced about the potential reasons for factor structure deviating from the original study. For one, the dropped item, not necessarily because of that single item but because the pre-processing steps seemed to have homogenized the items more than they originally were. Second, it seems that this entire procedure was based on the English version reported in the original study - however, the original version of the scale was in Korean. We therefore may have an issue of this being a translation of a translation - losing some information, context and differentiation between items in the process. Finally (but less likely), were there differences in inclusion criteria between the studies.

Supplement

This should have been done in the original paper as well - but do provide both the English version as you do AND the Persian version (the one actually administered).

Reviewer #2: The merit of the article is its translation and validation of a Persian version of the PCCS, based on exploratory and confirmatory factor analyses of responses from nurses. The contribution would be greater if a model with antecedents and/or consequences of the PCCS were tested, for example, to assess nomological validity and criterion validity.

[1] Unifatorial or tri-fatorial?

[1.1] Joo et al. (2024) developed a 12-item scale across three dimensions: information sharing (5 items), patient-as-person (4 items), and therapeutic alliance (3 items).

[1.2] Yan et al. (pre-print): three dimensions: (6 items), (3 items), and (2 items).

[1.3] Amin et al. (2025, p.3) (2.3.4) confirm the three-factor structure for the PCCS, but they did not present these results and in subsequent analyses, they used this scale as if it were unidimensional:

• a single Cronbach's alpha instead of a measure per factor.

• • Table 1: summated rating scale (range, minimum, etc.)

• • Table 3: correlation

Amin, S. M. et al. (2025). The moral middle ground: How moral distress mediates the link between patient-centered communication and palliative caring competence among oncology nurses. European Journal of Oncology Nursing, 79, 1-9. https://doi.org/10.1016/j.ejon.2025.103003

Yan, F., ru Wu, X., Yang, S., & Zhang, H. (2025). Psychometric Validation and Cross-Cultural Adaptation of the Chinese Patient-Centered Communication Scale for Nurses. Pre-print, without peer review. Research Square. https://doi.org/10.21203/rs.3.rs-7442607/v1

[1.4] Study under analysis: exploratory and confirmatory factor analysis, with unidimensional result and 11 items.

Suggestion 1: Discuss the issue of the dimensionality of the PCCS right at the beginning of the article. I suggest testing the model with 3 dimensions, as per Joo et al. (2024), through a confirmatory factor analysis. If the model is rejected, then proceed to exploratory analysis.

p.4) “it seems necessary to have an instrument that reflects this concept from the

93 viewpoint of nurses as HCPs, rather than patients and their families”

Comment: Just because it hasn't been done yet doesn't mean it needs to be done. I understand that the patient's and family's perspective is more important. Why would the nurse's point of view be important or necessary?

Suggestion: Justify this decision and provide an example.

p.13 and 15) “indicated normal univariate and multivariate distribution of the items, respectively.”

Comment: The items were measured on a 5-point scale, so it's not possible for the distribution to be normal, and the data are not even continuous. I suggest reviewing these comments.

p.34) What is the justification for including two correlations between the error terms (e2-e4) and (e9-e11)? If it was based solely on the modification indices, I suggest removing these arrows and re-estimating the model, or including a justification, even if it is post hoc.

p.35) The Appendix has the English version; I suggest including the Persian version as well, including the instructions given to the research participants (respondents).

Reviewer #3: The study aims to translate the Patient-Centered Communication Scale into Persian and to culturally adapt it to the Iranian context. The authors followed established guidelines for translation and cross-cultural adaptation and conducted appropriate statistical analyses to support the validation process. Overall, the methodological approach is sound and consistent with current standards in the field. However, the methods and discussion section require further development, and specific points for improvement are outlined below.

ABSTRACT

1. The type of sampling should be mentioned in the abstract.

2. The study assessed the validity of the scale among Iranian clinical nurses within specific medical facilities. Therefore, the generalizability of the findings is limited and should be explicitly acknowledged in the abstract, discussion, and conclusion sections. This issue is further elaborated upon in the comments on the discussion section.

3. The conclusion of the abstract could be strengthened by more clearly addressing the potential applications of the findings and explicitly stating what is being assessed. Additionally, caution should be exercised to avoid overgeneralization beyond the study context.

INTRODUCTION

1. The description of the original Patient-Centered Communication Scale (PCCS) should be refined, particularly regarding its validation study. Provide specifics on how the validation was conducted, the number of health professionals involved, the setting and timing, the psychometric features analyzed, and the factor structure.

METHODS

1. Step 4: Backward translation (line 151):

It should be stated if the two translators independently translated the Persian version of the PCCS back to English.

2. Exploratory factor analysis (line 230):

The maximum likelihood method assumes continuous variables, whereas PCCS items are ordinal (Likert-type scale from 1 to 5). Was an alternative extraction method suitable for categorical/ordinal data considered, such as Weighted Least Squares (WLSMV) or diagonally weighted least squares (DWLS)? This methodological choice, and its potential impact on factor structure and loadings, should be explicitly discussed in the limitations/discussion section.

3. Confirmatory factor analysis (line 238):

Numerous fit indices are reported, making the results presentation unnecessarily lengthy. I recommend focusing on the most standard metrics (e.g., CFI, TLI, RMSEA, SRMR) with established cutoffs and citing relevant references. This will streamline the presentation while maintaining methodological rigor.

4. Confirmatory factor analysis (line 238):

The estimator used should be specified and whether modification indices were examined.

5. Reliability (line 263):

The test-retest method employed a 14-day interval between data collections. Please provide justification for selecting this specific time frame, considering potential influences on score stability (e.g., memory effects with shorter intervals vs. true change with longer intervals).

6. Normal distribution and outlier values (line 266):

It should be presented why normality was evaluated and its relevance to the maximum likelihood estimator explicitly stated. Given the categorical/ordinal nature of the data, it should also be considered discussing sensitivity analyses or robust alternatives.

RESULTS

1. Some demographic variables should be better explained, such as income adequacy and ward. Income adequacy may be self-explanatory in the Iranian context, but it is not sufficiently clear for an international readership and should be defined in the manuscript. In addition, since nurses may work in more than one ward, as suggested by Table 1, this point should be clarified and consistently reported in the text.

2. Content validity (line 295):

When describing changes made to the items, it should be helpful to include the full item statements (both original and revised versions).

3. Construct validity (line 318):

The term “prove” should be avoided, as goodness-of-fit metrics indicate whether the covariance matrix predicted by the model is well supported by the observed data, rather than providing definitive proof.

4. Reliability (line 346):

All descriptive information regarding study procedures should be presented exclusively in the methods section to maintain consistent reporting structure throughout the manuscript.

DISCUSSION

1. Second paragraph (line 366):

The removal of an item represents a more substantial difference between the original and translated scale than the factor structure, as item removal may influence the factor structure itself. Therefore, it should be discussed first, before addressing the factor structure comparison.

2. Second paragraph (line 374):

The reasoning behind changes in factor structure between the original and translated versions may be multidimensional and difficult to definitively assess. Explanations should therefore be phrased suggestively (e.g., "may reflect," "could be attributed to") rather than affirmatively, acknowledging the complexity and potential multiple contributing factors.

3. Third paragraph (line 400):

MaxR provides an upper-bound estimate of reliability under optimal weighting of items. It does not mean MaxR is the “strongest type of reliability”. There is no universally best type of reliability. The most appropriate measure depends on the specific construct and context being evaluated.

4. Third paragraph (line 405):

Claims of "superiority" over other studies should be avoided. The original scale development study focused on establishing the initial scale, items, and foundational validation, which represents only one phase of the comprehensive scale development process. Improvements relative to prior studies should be highlighted constructively rather than through direct comparisons of importance.

5. A detailed limitations section should be added, essential for contextualizing the findings, acknowledging possible problems or vulnerabilities, and describing measures taken to address them. Key issues that must be discussed include: convenience sampling and its implications for generalizability; potential lack of independence among respondents (e.g., could participants have completed the survey collaboratively during spare time?); and the categorical nature of outcomes and its implications for maximum likelihood estimation assumptions.

6. The discussion did not sufficiently address differences in sample characteristics—both observable and unobservable between the original scale study sample and the current study sample. These differences should be explicitly discussed as potential influences on the observed results.

7. The discussion should include broader consideration of alternative scales. If no other patient-centered communication scales exist, relevant scales measuring patient or family perspectives should be presented, along with a balanced assessment of their relative advantages and disadvantages compared to the PCCS.

CONCLUSION

1. It is important to emphasize that the translated PCCS was validated among Iranian nurses in two very specific facilities. Claims about the generalizability of these results should be appropriately revised throughout the manuscript to present conclusions that accurately reflect this limited scope.

**********

what does this mean?). If published, this will include your full peer review and any attached files.

If you choose “no”, your identity will remain anonymous but your review may still be made public.

**Do you want your identity to be public for this peer review?**  For information about this choice, including consent withdrawal, please see our Privacy Policy

Reviewer #1: **Yes:** Marin Dujmović

Reviewer #2: **Yes:** Diogenes de Souza Bido

Reviewer #3: No

**********

[NOTE: If reviewer comments were submitted as an attachment file, they will be attached to this email and accessible via the submission site. Please log into your account, locate the manuscript record, and check for the action link "View Attachments". If this link does not appear, there are no attachment files.]

To ensure your figures meet our technical requirements, please review our figure guidelines: https://journals.plos.org/plosone/s/figures

You may also use PLOS’s free figure tool, NAAS, to help you prepare publication quality figures: https://journals.plos.org/plosone/s/figures#loc-tools-for-figure-preparation.

NAAS will assess whether your figures meet our technical requirements by comparing each figure against our figure specifications.

# Author comment: Author response to Decision Letter 1

**DOI:** 10.1371/journal.pone.0358575.r002

3 Jul 2026

Editor: PLOS One.

July 3, 2026

Dear Dr Maria José Nogueira;

Thank you very much for considering our revised manuscript, "Psychometric features of the Patient-Centered Communication Scale among Iranian clinical nurses". Manuscript Number: PONE-D-26-05593 for potential placement in PLOS One.

We have revised the manuscript in accordance with the editorial recommendations. Our responses are given in a point-by-point manner in the grid that is presented below. In the revised manuscript, we have highlighted the corrected text in accordance with the reviewers' and editor's recommendations.

We sincerely appreciate the opportunity to provide these revisions to the manuscript and believe the recommendations have continued to improve the paper. We hope the revised version is now suitable for publication and look forward to hearing from you in this regard.

Sincerely,

Journal Requirements

Comment: 1. Please ensure that your manuscript meets PLOS ONE's style requirements, including those for file naming. The PLOS ONE style templates can be found at

https://journals.plos.org/plosone/s/file?id=wjVg/PLOSOne_formatting_sample_main_body.pdf and

https://journals.plos.org/plosone/s/file?id=ba62/PLOSOne_formatting_sample_title_authors_affiliations.pdf

Response: The PLOS ONE journal style requirements, including file naming, were followed in accordance with the submitted links.

---

Comment: 2. In the online submission form you indicate that your data is not available for proprietary reasons and have provided a contact point for accessing this data. Please note that your current contact point is a co-author on this manuscript. According to our Data Policy, the contact point must not be an author on the manuscript and must be an institutional contact, ideally not an individual. Please revise your data statement to a non-author institutional point of contact, such as a data access or ethics committee, and send this to us via return email. Please also include contact information for the third-party organization, and please include the full citation of where the data can be found.

Response: A non-author institutional contact point, the Vice Chancellor for Research and Technology of Shahroud University of Medical Sciences, was added to the mentioned statement, along with an electronic address and telephone number for reasonable requests by researchers who meet the criteria for access to confidential data.

Therefore, the data availability statement was amended as follows:

"The data supporting the results of this study contain potentially personally identifying and sensitive information about the participants. Therefore, this data cannot be made publicly available according to the policies and regulations of the Research Ethics Committee of Shahroud University of Medical Sciences. The ethical approval of this study also explicitly prohibits public sharing of the data to protect the confidentiality and privacy of the participants. However, the data will be accessible upon reasonable request from the corresponding author via email (abbasi_msn@yahoo.com) or from the Vice President for Research and Technology of this university at the electronic address https://shmu.ac.ir/research/en and at the telephone number +982332396714 via email (vcr@shmu.ac.ir) to researchers who meet the criteria for access to confidential data."

---

Comment: 3. You have indicated that data is available from [vcr@shmu.ac.ir]. Please can we ask you to provide us with a general contact email address for the data requests, so readers can request access in perpetuity. If a general email is not available, please provide a link to a website where readers can obtain access to data.

Response: In addition to the aforementioned email, the electronic address and telephone number of the Vice Chancellor for Research and Technology of Shahroud University of Medical Sciences were also included in the mentioned statement for reasonable requests by researchers who meet the criteria for access to confidential data.

---

Comment: 4. Please upload a new copy of Figure 1 as the detail is not clear. Please follow the link for more information: https://journals.plos.org/plosone/s/figures

Response: Figure 1 was uploaded to the system in higher quality, in accordance with the PLOS ONE journal style requirements.

---

Comment: 5. If the reviewer comments include a recommendation to cite specific previously published works, please review and evaluate these publications to determine whether they are relevant and should be cited. There is no requirement to cite these works unless the editor has indicated otherwise.

Response: The articles that the reviewers introduced were not only fully relevant to the present manuscript but also helped to further enrich the "Discussion" and "Introduction" sections.

Only one of the articles was pre-printed and without peer review, which was excluded from citation in coordination with the respected reviewer.

---

Comment: 6. Please review your reference list to ensure that it is complete and correct. If you have cited papers that have been retracted, please include the rationale for doing so in the manuscript text, or remove these references and replace them with relevant current references. Any changes to the reference list should be mentioned in the rebuttal letter that accompanies your revised manuscript. If you need to cite a retracted article, indicate the article’s retracted status in the References list and also include a citation and full reference for the retraction notice.

Response: The list of references was re-examined.

While confirming its correctness and completeness, this manuscript does not cite any rejected articles.

---

Reviewer #1

Comment: The authors set out to translate the original PCC scale from Joo et al. (2024) to Persian and determine validity/reliability on an Iranian sample of nurses.

I found the paper very well written with a good level of detail but I also have a few fairly major issues with the approach.

Response: Thank you for your encouraging words.

It is hoped that the following amendments will enhance the quality of this manuscript.

---

Comment: The biggest issue is removing item 4 from the original paper (actually item 1 as numbered in Table 2 of the original paper (though it does appear 4th from top in that table). The authors describe a detailed procedure of iterative translation, evaluation and modification of the scale. I fear that the good intention of being thorough has resulted in issues due to too much "pre-processing".

Namely, the original (well, at least the original translation from Korean to English) versions of the two items deemed redundant by the authors are as follows:

Item 1: "Describe the medical treatment/procedure or nursing intervention."

Item 2: "Describe the reason for the necessity of medical treatment/procedures or nursing intervention."

The first is about WHAT is being done and the second WHY it is being done. The distinction is quite clear, and although I am not fluent in Persian, I believe it should have been straightforward to translate as such, especially considering the detailed procedure of translating the original.

This indicates that the lengthy pre-processing stage itself, at least in part, homogenized the items. If it made these two items so similar that one was deemed redundant then it may have made the rest more similar and therefore contributed to the strong unidimensional structure when compared to the 3-factor solution of the original.

I am also concerned that such a detailed procedure could not result in this simple distinction between WHAT and WHY as quite a simple one.

Response: We sincerely thank you for your beneficial perspective.

The authors fully agree with your view on the conceptual distinction between "what is done" and "why it is done".

However, it is emphasized that the conceptual overlap observed between these two items is not due to their simplification or homogenization in the process of translation and cultural adaptation, because in all stages of the model proposed by Wild et al. (2005), an attempt was made to maintain the conceptual distinction between what and why.

The expert panel in the present study reported significant conceptual overlap between the two items because from their perspective, in everyday nurse-patient interactions, explaining the type and nature of care or treatment is usually accompanied by explaining its cause and necessity. In fact, these two communication behaviors rarely occur independently of each other in such a way that their practical separation seems difficult in many clinical situations. As a result, both items were considered to reflect a common aspect of patient-centered communication, namely, providing information and explaining care or treatment to the patient or their families.

Accordingly, both the "Discussion" section and the "Content validity" section of the results were completely revised to explain the deletion of item 4 with greater clarity.

It is worth noting that it is explicitly stated in the "Discussion" section that the two items are not entirely identical from a theoretical perspective. It is also suggested that future research examine the retention of the two items and their performance in subsequent psychometric stages in independent samples and in different contexts.

The changes have been highlighted in yellow (Pages 18 and 19; Lines 376-395; Results section and Pages 23-25; Lines 478-509; Discussion section).

---

Comment: I have two further approach-related issues. First, the confirmatory analysis should have compared the unidimensional model to a 3-factor model as proposed by the original paper. From what I can see, it is almost a certainty that absolutely nothing will change and that a unidimensional structure fits better. However, I would still advise doing it as the original paper provides one hypothesis and the first wave of data collection with the adopted version another - these can be neatly compared. And second, I strongly disagree with data not being made available. This is not patient data, and it can be sufficiently anonymized. I would have considered uploading the questionnaire data alongside perhaps age, sex and total work experience.

Response: We thank the reviewer for these valuable methodological suggestions.

We have now added a formal comparison between the unidimensional and the original three factor model using the chi square difference test, ΔCFI, AIC, and BIC. The results confirm that the unidimensional model is more parsimonious and not significantly worse than the three factor solution.

The changes have been highlighted in yellow (Pages 13-15; Lines 281-319; Methods section and Pages 19-22; Lines 396-445; Results section).

This data cannot be made publicly available according to the policies and regulations of the Research Ethics Committee of Shahroud University of Medical Sciences. However, the data will be accessible upon reasonable request from the corresponding author via email (abbasi_msn@yahoo.com) or from the Vice President for Research and Technology of this university at the electronic address https://shmu.ac.ir/research/en and at the telephone number +982332396714 via email (vcr@shmu.ac.ir) to researchers who meet the criteria for access to confidential data.

These details are also provided in the data availability statement.

---

Comment: Considering that I believe that the process by which the items were translated and modified has contributed to the homogeneity of the factor structure - the recommendation is trickier than it might seem. On one hand, the paper is very well written, and the authors have obviously been thorough. Additionally, the scale itself is likely to be useful for researchers from Iran and beyond. On the other hand, the big issue being the only part of the process that cannot be addressed (the pre data collection stage) makes it problematic.

I am suggesting major revisions, if the authors make changes to, primarily, how they discuss differences between the original and their own version (see comments below). I believe this can be published, with some very obvious caveats.

See specific issues for each section below.

Response: The "Discussion" section has been completely revised to not only address your valuable concerns but also to justify the deletion of item 4 in the content validity stage and the difference in the dimensionality of the original and Persian versions of the Patient-Centered Communication Scale from various perspectives.

The changes have been highlighted in yellow (Pages 23-26; Lines 478-551; Discussion section).

---

Introduction

Comment: Lines 84-92 – authors give the argument that the scales usually being used were not designed with nurses in mind – they then mention that others have explored validity and psychometric properties of other scales – but not what the findings were and how that informs their decision. Perhaps there's a bit of a language barrier here as in the examples were meant to enumerate that work has been done but not from nurses' perspective and nothing more. As currently written, it feels like there is more to the argument, something about the properties of these scales which is not mentioned.

Response: Thank you for your constructive feedback.

Other instruments that assess this concept from the perspective of patients and their families, including the Patient-Centered Communication Instrument (PCCI) and the Caregiver-Centered Communication Questionnaire (CCCQ), were presented, with emphasis on psychometric properties, number of items, subscales measured, and target population.

Additionally, the importance of having an instrument that measures patient-centered communication from the perspective of nurses as the primary healthcare providers was discussed in detail.

The changes have been highlighted in yellow (Pages 4 and 5; Lines 84-119; Introduction section).

---

Comment: Line 95 – should not have the year in parentheses as it isn’t consistent with the referencing style used here.

Response: Thank you for your attention to detail.

The mentioned item was corrected.

---

Methods

Comment: Line 115-117 – it is not clear why an undergraduate degree and a year of experience was required for taking part in the study – surely that reduces the possible range of results compared to how it the scale may be used.

Response: A bachelor of science in nursing or higher and a minimum of one year of full-time clinical experience were selected, in line with previous psychometric studies in Iranian clinical nurses.

• Jalali A, Sharifi A, Babaei K, Moradi K, Karimi J, Moradi L, et al. Psychometric properties of the Persian version of the Nursing Work Environment Scale during public health emergencies. Critical Public Health. 2026;36(1):2683748. doi: 10.1080/09581596.2026.2683748.

• Jafari Rahim Abad Y, Saadati K, Ghaffari F. Psychometric evaluation of the ethical caring competency scale among Iranian Nurses: A methodological study. PLOS ONE. 2026;21(5):e0346672. doi: 10.1371/journal.pone.0346672.

• Tehranineshat B, Rivaz M, Kargar Dolatabadi E. Psychometric testing of the Persian version of the Nursing Care Quality Scale: A methodological study. Nurs Open. 2023;10(9):6491-500. doi: 10.1002/nop2.1900.

In addition, the following sentences were added to the "Inclusion and exclusion criteria" section of the method to justify the selection of the aforementioned inclusion criteria.

"A minimum of one year of full-time clinical experience not only provides sufficient exposure to care situations and patient-centered interactions but also increases the likelihood of accurately comprehending the items based on actual clinical experience. On the other hand, a bachelor of science in nursing or higher, as the minimum level of professional education in many nursing systems, including Iran, facilitates understanding and interpretation of the content of the items. These criteria were selected in line with previous psychometric studies in Iranian clinical nurses [18-20]."

The changes have been highlighted in yellow (Pages 7 and 8; Lines 160-169; Methods section).

---

Comment: Line 264-265 – how were the nurses for test-retest selected? Was this random? If not, it could have biased the estimate.

Response: Thank you for your constructive feedback.

Participants in this phase were randomly selected with maximum diversity in demographic characteristics.

These details have been added to the "Reliability" section of the methods.

The changes have been highlighted in yellow (Pages 15 and 16; Lines 331-342; Methods section).

---

Results

Comment: Table 1 – being too pedantic here – but I would stick to two decimal spots for the % to keep in line with the rest of the paper (even if the value is something like 26.70).

Response: All percentages, whether in tables or text, were reported to two decimal places.

---

Comment: Figure 1 shows that residuals of Item 11&12 as well as 6&7 were allowed to correlate. This was not mentioned in text, what was the criteria by which this was decided? Were modification indices looked at diagnostically then a decision made based on something like method effects due to similar wording? I do think, looking at the content, that the decision is fine - it should just not be glossed over. I would report model indices with and without the modification (perhaps the initial model fit can be a table in an appendix / supplement to keep the main text flow).

Response: We thank the reviewer for this careful observation.

We agree that the residual correlations in Figure 1 should not have been left unexplained.

We have now explicitly added to the Methods that modification indices were inspected, and residual covariances were added only when the modification indices exceeded 10 and the correlation was theoretically justifiable (shared content on interdisciplinary collaboration for items 11 & 12 and shared content on emotional expression for items 6 & 7).

In the Results, we now report the initial model fit (without correlations), the modification indices values, the theoretical rationale, and the improvement after adding the two covariances.

The changes have been highlighted in yellow (Pages 14 and 15; Lines 298-319; Methods section and Page 21; Lines 425-440; Results section).

---

Discussion

Comment: Considering the issues I mention above, I believe the discussion should be more nuanced about the potential reasons for factor structure deviating from the original study. For one, the dropped item, not necessarily because of that single item but because the pre-processing steps seemed to have homogenized the items more than they originally were. Second, it seems that this entire procedure was based on the English version reported in the original study - however, the original version of the scale was in Korean. We therefore may have an issue of this being a translation of a translation - losing some information, context and differentiation between items in the process. Finally (but less likely), were there differences in inclusion criteria between the studies.

Response: Possible reasons for the difference in the dimensionality of the original and Persian versions of the Patient-Centered Communication Scale, including some apparent differences between the demographic characteristics of the participants in the two studies; the decision to delete an item at the content validity stage; the use of the English version provided by the original designer as the basis for translation; personal or behavioral differences between Korean and Iranian clinical nurses; as well as the different conditions prevailing in the healthcare systems of the two countries, were detailed in the "Discussion" section.

The changes have been highlighted in yellow (Pages 25 and 26; Lines 510-551; Discussion section).

---

Supplement

Comment: This should have been done in the original paper as well - but do provide both the English version as you do AND the Persian version (the one actually administered).

Response: Both the Persian and English versions of the data collection instruments, including the demographic characteristics checklist, as well as the Persian version of the Patient-Centered Communication Scale, after going through all the stages of cultural adaptation and psychometric evaluation, have been uploaded as supplementary files.

---

Reviewer #2

Comment: The merit of the article is its translation and validation of a Persian version of the PCCS, based on exploratory and confirmatory factor analyses of responses from nurses. The contribution would be greater if a model with antecedents and/or consequences of the PCCS were tested, for example, to assess nomological validity and criterion validity.

Response: Thank you for your encouraging words.

Assessment of nomological validity and criterion validity were mentioned as suggestions for future research to enhance the research and clinical applications of this scale.

The changes have been highlighted in yellow (Page 29; Lines 618-630; Discussion section).

---

Comment: Unifactorial or tri-factorial?

[1.1] Joo et al. (2024) developed a 12-item scale across three dimensions: information sharing (5 items), patient-as-person (4 items), and therapeutic alliance (3 items).

[1.2] Yan et al. (pre-print): three dimensions: (6 items), (3 items), and (2 items).

[1.3] Amin et al. (2025, p.3) (2.3.4) confirm the three-factor structure for the PCCS, but they did not present these results and in subsequent analyses, they used this scale as if it were unidimensional:

• a single Cronbach's alpha instead of a measure per factor.

• Table 1: summated rating scale (range, minimum, etc.)

• Table 3: correlation

Amin, S. M. et al. (2025). The moral middle ground: How moral distress mediates the link between patient-centered communication and palliative caring competence among oncology nurses. European Journal of Oncology Nursing, 79, 1-9. https://doi.org/10.1016/j.ejon.2025.103003

[1.4] Yan, F., ru Wu, X., Yang, S., & Zhang, H. (2025). Psychometric Validation and Cross-Cultural Adaptation of the Chinese Patient-Centered Communication Scale for Nurses. Pre-print, without peer review. Research Square. https://doi.org/10.21203/rs.3.rs-7442607/v1

Study under analysis: exploratory and confirmatory factor analysis, with unidimensional result and 11 items.

Discuss the issue of the dimensionality of the PCCS right at the beginning of the article.

Response: The dimensionality and three-factor structure of the Patient-Centered Communication Scale based on the study by Joo et al. (2024) and Amin et al. (2025) were discussed at the end of the "Introduction" section.

In addition, the article by Yan et al. was not cited because it was preprinted and not peer-reviewed.

The changes have been highlighted in yellow (Pages 6 and 7; Lines 120-148; Introduction section).

---

Comment: I suggest testing the model with 3 dimensions, as per Joo et al. (2024), through a confirmatory factor analysis. If the model is rejected, then proceed to exploratory analysis.

Response: We thank the reviewer for these valuable methodological suggestions.

We have now restructured the entire factor analysis section to strictly follow the recommended sequence: the original three factor model was first tested via CFA and rejected due to inadequate fit and poor discriminant validity. Only then did we proceed with EFA to explore the underlying structure, and the resulting unidimensional model was subsequently confirmed by CFA on the independent sample. This revised order is now clearly presented in both the Methods and Results sections, with the initial three factor fit indices reported upfront. We believe this addresses the reviewer’s concern comprehensively.

The changes have been highlighted in yellow (Pages 13-15; Lines 281-319; Methods section and Pages 19-22; Lines 396-445; Results section).

---

Comment: p.4) “it seems necessary to have an instrument that reflects this concept from the viewpoint of nurses as HCPs, rather than patients and their families”

Just because it hasn't been done yet doesn't mean it needs to be done. I understand that the patient's and family's perspective is more important. Why would the nurse's point of view be important or necessary?

Justify this decision and provide an example.

Response: The importance of having an instrument that measures patient-centered communication from the perspective of nurses as the primary healthcare providers was discussed in detail.

The aforementioned paragraph, while providing illustrative examples, was written with the focus on the complementarity of the perspectives of nurses and the perspectives of patients and their families to form a more multidimensional understanding of this concept in clinical settings.

The changes have been highlighted in yellow (Page 5; Lines 97-119; Introduction section).

---

Comment: p.13 and 15) “indicated normal univariate and multivariate distribution of the items, respectively.”

The items were measured on a 5-point scale, so it's not possible for the distribution to be normal, and the data are not even continuous. I suggest reviewing these comments.

Response: We thank the reviewer for this careful observation.

We fully agree that individual Likert-scale items are ordinal and cannot be strictly normally distributed. However, in psychometric practice, the reported skewness and kurtosis values (within ±3 and ±7, respectively) as well as the Mardia coefficient (<8) were not used to claim mathematical normality of the raw ordinal responses. Rather, these indices were applied to assess approximate univariate and multivariate normality of the item distributions—a routine and widely accepted step to justify the use of maximum likelihood estimation in confirmatory factor analysis. Maximum likelihood estimation is known to be robust to moderate deviations from normality with five-point Likert data, provided that skewness and kurtosis fall within these conventional cut-offs.

The changes have been highlighted in yellow (Page 16; Lines 343-353; Methods section).

---

Comment: p.34) What is the justification for including two correlations between the error terms (e2-e4) and (e9-e11)? If it was based solely on the modification indices, I suggest removing these arrows and re-estimating the model, or including a justification, even if it is post hoc.

Response: The theoretical justification for these residual correlations (based on content overlap and shared wording) has now been explicitly added to the Methods and Results sections of the revised manuscript.

The changes have been highlighted in yellow (Pages 14 and 15; Lines 298-319; Methods section and Page 21; Lines 425-440; Results section).

---

Comment: p.35) The Appendix has the English version; I suggest including the Persian version as well, including the instructions given to the research participants (respondents).

Response: Both the Persian and English versions of the data collection instruments, including the demographic characteristics checklist, as well as the Persian version of the Patient-Centered Communication Scale, after going through all the stages of cultural adaptation and psychometric evaluation, have been uploaded as supplementary files.

---

Reviewer #3

Comment: The study aims to translate the Patient-Centered Communication Scale into Persian and to culturally adapt it to the Iranian context. The authors followed established guidelines for translation and cross-cultural adaptation and conducted appropriate statistical analyses to support the validation process. Overall, the methodological approach is sound and consistent with current standards in the field. However, the methods and discussion section require further development, and specific points for improvement are outlined below.

Response: Thank you for your encouraging words.

It is hoped that the following amendments will enhance the quality of this manuscript.

---

ABSTRACT

Comment: 1. The type of sampling should be mentioned in the abstract.

Response: The sampling approach used was described in the "Methods" section of the abstract.

The changes have been highlighted in yellow (Page 2; Lines 27-35; Abstract section).

---

Comment: 2. The study assessed the validity of the scale among Iranian clinical nurses within specific medical facilities. Therefore, the generalizability of the findings is limited and should be explicitly acknowledged in the abstract, discussion, and conclusion sections. This issue is further elaborated upon in the comments on the discussion section.

Response: Not only was the single-center data collection from clinical nurses at hospitals associated with Shahroud University of Medical Sciences (Imam Hossein and Bahar Hospitals) discussed in both the abstract and the conclusion, but other limitations that may affect the generalizability of the findings were also included in a new section titled "Limitations, strengths, and suggestions".

The changes have been highlighted in yellow (Pages 2 and 3; Lines 42-47; Abstract section and Page 29; Lines 607-617; Discussion section and Page 30; Lines 634-640; Conclusion section).

---

Comment: 3. The conclusion of the abstract could be strengthened by more clearly addressing the potential applications of the findings and explicitly stating what is being assessed. Additionally, caution should be exercised to avoid overgeneralization beyond the study context.

Response: The "Conclusion" section of the abstract discussed the potential applications of the Persian version of this scale, as well as the limitations surrounding the generalizability of the findings.

The changes have been highlighted in yellow (Pages 2 and 3; Lines 42-47; Abstract section).

---

INTRODUCTION

Comment: 1. The description of the original Patient-Centered Communication Scale (PCCS) should be refined, particularly regarding its validation study. Provide specifics on how the validation was conducted, the number of health professionals involved, the setting and timing, the psychometric features analyzed, and the factor structure.

Response: Thank you for your valuable comment.

The description of the Patient-Centered Communication Scale in the "Introduction" section has been revised in general.

Additionally, the details of the step-by-step and systematic approach that Joo et al. (2024) took to obtain a valid and reliable instrument for measuring this concept from the perspective of nurses, who are the main healthcare providers, have been thoroughly described.

These details included how to assess face validity, content validity, construct validity through exploratory factor analysis and confirmatory factor analysis, convergent validity, and internal consistency.

The changes have been highlighted in yellow (Page 6; Lines 120-133; Introduction section).

---

METHODS

Comment: 1. Step 4: Backward translation (line 151):

It should be stated if the two translators independently translated the Persian version of the PCCS back to English.

Response: Thank you for your attention to detail.

The mentioned item was corrected.

---

Comment: 2. Exploratory factor analysis (line 230):

The maximum likelihood method assumes continuous variables, whereas PCCS items are ordinal (Likert-type scale from 1 to 5). Was an alternative extraction method suitable for categorical/ordinal data considered, such as Weighted Least Squares (WLSMV) or diagonally weighted least squares (DWLS)? This methodological choice, and its potential impact on factor structure and loadings, should be explicitly discussed in the limitations/discussion section.

Response: We thank the reviewer for these valuable methodological suggestions.

We fully acknowledge that Patient-Centered Communication Scale items are ordinal (five point Likert scale) and that strictly speaking, they are not continuous. However, in psychometric practice, the use of maximum likelihood estimation with ordinal data—particularly when the scale has five or more response categories—is widely accepted and has been shown to produce reliable parameter estimates, factor loadings, and model fit indices, provided that the data do not exhibit severe violations of normality. In our study, the skewness and kurtosis values for all items fell well within the conventional ranges of ±3 and ±7, respectively, and the Mardia coefficient was below 8, indicating acceptable approximate univariate and multivariate normality. These conditions justify the use of maximum likelihood estimation.

The changes have been highlighted in yellow (Page 16; Lines 343-353; Methods section).

Moreover, maximum likelihood estimation is robust to moderate departures from normality when the sample size is adequate (n=150 per analysis), which is the case in our study. While alternative estimators such as Weighted Least Squares (WLSMV) or diagonally weighted least squares (DWLS) are specifically designed for categorical data, they often require larger sample sizes and may be overly conservative with five point Likert items that behave similarly to continuous variables. Given the satisfactory normality indicators and the established precedent in the psychometric literature, we chose maximum likelihood estimation as a well balanced and appropriate approach for our factor analyses. We have now added a brief clarification in the "Limitations, strengths, and suggestions" section to acknowledge this methodological choice and its rationale.

The changes have been highlighted in yellow (Page 29; Lines 618-630; Discussion section).

---

Comment: 3. Confirmatory factor analysis (line 238):

Numerous fit indices are reported, making the results presentation unnecessarily lengthy. I recommend focusing on the most standard metrics (e.g., CFI, TLI, RMSEA, SRMR) with established cutoffs and citing relevant references. This will streamline the presentation while maintaining methodological rigor.

Response: Only two indices from each group of fit indices, namely absolute (χ²/df and RMSEA), comparative (CFI and TLI), and parsimonious (PNFI and PCFI), were reported in order to present the results more simply while maintaining methodological rigor.

---

Comment: 4. Confirmatory factor analysis (line 238):

The estimator used should be specified and whether modification indices were examined.

Response: We have now explicitly specified that maximum likelihood estimation was used for all CFA models. We also confirmed that modification indices were examined to guide residual covariances, as already reported in the Results. The corresponding sentence has been added to the Methods section (Confirmatory factor analysis).

The changes have been highlighted in yellow (Pages 14 and 15; Lines 298-319; Methods section).

---

Comment: 5. Reliability (line 263):

The test-retest method employed a 14-day interval between data collections. Please provide justification for selecting this specific time frame, considering potential influences on score stability (e.g., memory effects with shorter intervals vs. true change with longer intervals).

Response: Thank you for your valuable comment.

The following statements have been added to the "Reliability" section of the methods to justify the choice of the mentioned time interval.

"The mentioned time interval was chosen in accordance with the methodological recommendations of psychometric studies. While it is long enough to reduce the likelihood of participants recalling previous responses, it is short enough to prevent real changes in perceptions of patient-centered communication [41, 42]."

The changes have been highlighted in yellow (Pages 15 and 16; Lines 331-342; Methods section).

---

Comment: 6. Normal distribution and outlier values (line 266):

It should be presented why normality was evaluated and its relevance to the maximum likelihood estimator explicitly stated. Given the categorical/ordinal nature of the data, it should also be considered discussing sensitivity analyses or robust alternatives.

Response: We thank the reviewer for this careful observation.

We have now explicitly stated in the "Normal distribution and outlier values" subsection why normality was evaluated—namely, to justify the use of the maximum likelihood estimator.

We also clarified that, despite the ordinal nature of the data, the approximate normality and adequate sample size support the maximum likelihood approach, and we noted that robust alternatives could be considered in future studies.

The changes have been highlighted in yellow (Page 16; Lines 343-353; Methods section and Page 29; Lines 618-630; Discussion section).

---

RESULTS

Comment: 1. Some demographic variables should be better explained, such as income adequacy and ward. Income adequacy may be self-explanatory in the Iranian context, but it is not sufficiently clear for an international readership and should be defined in the manuscript. In addition, since nurses may work in more than one ward, as suggested by Table 1, this point should be clarified and consistently reported in the text.

Response: The "Demographic characteristics checklist" section of the methods not only provided details about the department of practice but also defined income adequacy.

Additionally, the classification of the department of practice was also mentioned consistently throughout the manuscript.

The changes have been highlighted in yellow (Page 8; Lines 178-187; Methods section).

---

Comment: 2. Content validity (line 295):

When describing changes made to the items, it should be helpful to include the full item statements (both original and revised versions).

Response: The changes made in the content validity stage were presented with full mention of the desired items (both the original version and the Persian version).

The changes have been highlighted in yellow (Pages 18 and 19; Lines 376-395; Results section).

---

Comment: 3. Construct validity (line 318):

The term “prove” should be avoided, as goodness-of-fit metrics indicate whether the covariance matrix predicted by the model is well supported by the observed data, rather than providing definitive proof.

Response: Thank you for your attention to detail.

The word "suggest" has been replaced with "prove".

---

Comment: 4. Reliability (line 346):

All descriptive information regarding study procedures should be presented exclusively in the methods section to maintain consistent reporting structure throughout the manuscript.

Response: Descriptive information about participants in the external stability phase was moved from the "Reliability" section of the results to the "Reliability" section of the methods.

The changes have been highlighted in yellow (Pages 15 and 16; Lines 331-342; Methods section and Page 23; Lines 457-464; Results section).

---

DISCUSSION

Comment: 1. Second paragraph (line 366):

The removal of an item represents a more substantial difference between the original and translated scale than the factor structure, as item removal may influence the factor structure itself. Therefore, it should be discussed first, before addressing the factor structure comparison.

Response: We sincerely thank you for your beneficial perspective.

First, the deletion of item 4 in the content validity stage was discussed in detail.

Then, the difference in the dimensionality of the original and Persian versions of the Patient-Centered Communication Scale was justified from various perspectives.

The changes have been highlighted in yellow (Pages 23-25; Lines 478-509; Discussion section).

---

Comment: 2. Second paragraph (line 374):

The reasoning behind changes in factor structure between the original and translated versions may be multidimensional and difficult to definitively assess. Explanations should therefore be phrased suggestively (e.g., "may reflect," "could be attributed to") rather than affirmatively, acknowledging the complexity and potential multiple contributing factors.

Response: All explanations were given suggestively rather than affirmatively, because the difference in the dimensionality of the original and Persian versions of the Patient-Centered Communication Scale can be assessed in many ways, and making a definitive decision about it is not so simple.

---

Comment: 3. Third paragraph (line 400):

MaxR provides an upper-bound estimate of reliability under optimal weighting of items. It does not mean MaxR is the “strongest type of reliability”. There is no universally best type of reliability. The most appropriate measure depends on the specific construct and context being evaluated.

Response: The phrase "as it is the strongest type of reliability" was removed from the aforementioned paragraph.

The changes have been highlighted in yellow (Pages 27 and 28; Lines 582-594; Discussion section).

---

Comment: 4. Third paragraph (line 405):

Claims of "superiority" over other studies should be avoided. The original scale development study focused on establishing the initial scale, items, and foundational validation, which represents only one phase of the comprehensive scale development process. Improvements relative to prior studies should be highlighted constructively rather than through direct comparisons of importance.

Response: Thank you for your constructive feedback.

The sentence "distinguish the present study from the initial study of scale design" and the phrase "limitations of the initial study of scale design" were replaced with "demonstrate the superiority of the present study over the initial study of scale design" and "shortcomings of the initial study of scale design", respectively.

These changes not only avoided claims of superiority but also constructively highlighted improvements over other studies.

In addition, the advantages of McDonald’s omega coefficients compared to Cronbach’s alpha coefficients were more fully noted.

The changes have been highlighted in yellow (Pages 27 and 28; Lines 582-594; Discussion section).

---

Comment: 5. A detailed limitations section should be added, essential for contextualizing the findings, acknowledging possible problems or vulnerabilities, and describing measures taken to address them. Key issues that must be discussed include: convenience sampling and its implications for generalizability; potential lack of independence among respondents (e.g., could participants have completed the survey collaboratively during spare time?); and the categorical nature of outcomes and its implications for maximum likelihood estimation assumptions.

Response: Thank you for your valuable comment.

Selection bias due to the convenience sampling approach, the potential reduction in participant autonomy, and the subsequent emergence of biases such as group conformity or social desirability bias, as well as the five-point ordinal nature of the items and considering the alternative estimators specifically designed for categorical/ordinal data, has been addressed in a new section titled "Limitations, strengths, and suggestions".

The changes have been highlighted in yellow (Pages 29 and 30; Lines 607-633; Discussion section).

---

Comment: 6. The discussion did not sufficiently address differences in sample characteristics—both observable and unobservable between the original scale study sample and the current study sample. These differences should be explicitly discussed as potential influences on the observed results.

Response: We sincerely thank you for your beneficial perspective.

Observed and unobserved differences between the participants of the present study and the initial study of scale design were discussed in detail as possible reasons for the difference in the dimensionality of the original and Persian versions of the Patient-Centered Communication Scale.

The changes have been highlighted in yellow (Pages 25 and 26; Lines 510-551; Discussion section).

---

Comment: 7. The discussion should include broader consideration of alternative scales. If no other patient-centered communication scales exist, relevant scales measuring patient or family perspectives should be presented, along with a balanced assessment of their relative advantages and disadvantages compared to the PCCS.

Response: A more extensive review of existing instruments, including instruments based on the perspective of clinical nurses (PCCS) and instruments based on the perspective of patients or family caregivers (PCCI and CCCQ), along with a balanced presentation of the advantages and disadvantages of each relative to the Patient-Centered Communication Scale, is provided in the "Discussion" section.

The changes have been highlighted in yellow (Pages 26 and 27; Lines 552-575; Discussion section).

---

CONCLUSION

Comment: 1. It is important to emphasize that the translated PCCS was validated among Iranian nurses in two very specific facilities. Claims about the generalizability of these results should be appropriately revised throughout the manuscript to present conclusions that accurately reflect this limited scope.

Response: The single-center data collection from clinical nurses at hospitals associated with Shahroud University of Medical Sciences (Imam Hossein and Bahar Hospitals) was discussed in both the abstract and the conclusion to clearly reflect the limited scope of generalizability of the results.

Other limitations that may affect the generalizability of the findings are also included in a new section titled "Limitations, strengths, and suggestions".

The changes have been highlighted in yellow (Pages 2 and 3; Lines 42-47; Abstract section and Page 29; Lines 607-617; Discussion section and Page 30; Lines 634-640; Conclusion section).

Attachment Submitted filename: Response to Reviewers.pdf

# Peer review: Decision Letter 1

**DOI:** 10.1371/journal.pone.0358575.r003

**Authors:** Maria José Nogueira

23 Jul 2026

Dear Dr. Ghasempour,

Thank you for submitting your manuscript to PLOS ONE. After careful consideration, we feel that it has merit but does not fully meet PLOS ONE’s publication criteria as it currently stands. Therefore, we invite you to submit a revised version of the manuscript that addresses the points raised during the review process.

Please submit your revised manuscript by Sep 06 2026 11:59PM. If you will need more time than this to complete your revisions, please reply to this message or contact the journal office at plosone@plos.org. When you're ready to submit your revision, log on to https://www.editorialmanager.com/pone/ and select the 'Submissions Needing Revision' folder to locate your manuscript file.

- A letter that responds to each point raised by the academic editor and reviewer(s). You should upload this letter as a separate file labeled 'Response to Reviewers'.
- A marked-up copy of your manuscript that highlights changes made to the original version. You should upload this as a separate file labeled 'Revised Manuscript with Track Changes'.
- An unmarked version of your revised paper without tracked changes. You should upload this as a separate file labeled 'Manuscript'.

If you would like to make changes to your financial disclosure, please include your updated statement in your cover letter. Guidelines for resubmitting your figure files are available below the reviewer comments at the end of this letter.

If applicable, we recommend that you deposit your laboratory protocols in protocols.io to enhance the reproducibility of your results. Protocols.io assigns your protocol its own identifier (DOI) so that it can be cited independently in the future. For instructions see: https://journals.plos.org/plosone/s/submission-guidelines#loc-laboratory-protocols. Additionally, PLOS ONE offers an option for publishing peer-reviewed Lab Protocol articles, which describe protocols hosted on protocols.io. Read more information on sharing protocols at https://plos.org/protocols?utm_medium=editorial-email&utm_source=authorletters&utm_campaign=protocols.

As the corresponding author, your ORCID iD is verified in the submission system and will appear in the published article. PLOS supports the use of ORCID, and we encourage all coauthors to register for an ORCID iD and use it as well. Please encourage your coauthors to verify their ORCID iD within the submission system before final acceptance, as unverified ORCID iDs will not appear in the published article. *Only*  the individual author can complete the verification step; PLOS staff *cannot*  verify ORCID iDs on behalf of authors.

We look forward to receiving your revised manuscript.

Kind regards,

Maria José Nogueira, Ph.D.

Academic Editor

PLOS One

Journal Requirements:

If the reviewer comments include a recommendation to cite specific previously published works, please review and evaluate these publications to determine whether they are relevant and should be cited. There is no requirement to cite these works unless the editor has indicated otherwise.

Please review your reference list to ensure that it is complete and correct. If you have cited papers that have been retracted, please include the rationale for doing so in the manuscript text, or remove these references and replace them with relevant current references. Any changes to the reference list should be mentioned in the rebuttal letter that accompanies your revised manuscript. If you need to cite a retracted article, indicate the article’s retracted status in the References list and also include a citation and full reference for the retraction notice.

Additional Editor Comments :

The authors have revised the manuscript in accordance with the recommendations from the previous review round, and the overall quality of the manuscript has improved substantially.

However, several issues identified during the initial review remain insufficiently addressed and require further clarification before the manuscript can be considered for acceptance.

The authors are therefore requested to carefully address the additional comments provided by the reviewers and revise the manuscript accordingly.

[Note: HTML markup is below. Please do not edit.]

Reviewers' comments:

Reviewer's Responses to Questions

**Comments to the Author**

Reviewer #1: All comments have been addressed

Reviewer #2: All comments have been addressed

**********

2. Is the manuscript technically sound, and do the data support the conclusions?

Reviewer #1: Yes

Reviewer #2: Yes

**********

3. Has the statistical analysis been performed appropriately and rigorously?

Reviewer #1: Yes

Reviewer #2: Yes

**********

4. Have the authors made all data underlying the findings in their manuscript fully available?

The PLOS Data policy

Reviewer #1: Yes

Reviewer #2: No

**********

5. Is the manuscript presented in an intelligible fashion and written in standard English?

Reviewer #1: Yes

Reviewer #2: Yes

**********

Reviewer #1: The authors have responded to comments from myself and the other reviewers thoroughly and I believe the manuscript can get accepted with very minor edits..

One suggestion would be to split the data presented in Table 1 so that the two independently drawn samples of 150 nurses can be easily compared rather than collapsed across. They could even be formally compared via stats.

Second, Table 3 shows that the unidimensional model without correlating residuals provided a substantially worse fit than the three-factor model. But then only the unidimensional model was afforded the correlated residuals. I expect nothing would change as the unidimensional solution would remain more parsimonious - but the same affordance should be made for the three-factor model (of course if it was the case that MI-s did not exceed the threshold the authors identified then this is fine).

Third, now that I've revisited the scale, it occurs to me that all of the items are in the same direction and so straight-line responding is a possibility. It is also difficult to control for or identify as it would not be unreasonable to expect the same response on all items.

Fourth, it occurs to me that the mean, median, SD, range and other descriptive parameters for the total scale score were not reported anywhere. I'd suggest doing so for both of the samples only for the unidimensional solution. This is useful data to people considering the scale.

Finally, in the discussion - is there a reason to assume that relationship status would / should impact this scale? If not, I'd drop that bit. The big difference in work experience however is a good addition.

Reviewer #2: [1] Unifatorial or tri-fatorial?

Comments: Ok

[2] viewpoint of nurses

Comments: Ok

[3] normal X “robust to moderate violations of normality”

>> 1st submission - p.13 and 15) “indicated normal univariate and multivariate distribution of the items, respectively.”

>> R1 - p.16) “Skewness values spanning -3.00 to +3.00 and kurtosis values spanning -7.00 to +7.00, as well as Mardia coefficient values less than 8.00, indicated normal univariate and multivariate distribution of the items, respectively.”

Comments:

OK, “ML estimation is robust to moderate violations of normality”

But we cannot conclude that these items have a normal distribution.

Suggestion: Remove this sentence or cite methodological authors that justify this statement.

[4] Correlations between the error terms

Comments: Ok

[5] Persian version as supplementary files.

Comments: Ok

**********

what does this mean?). If published, this will include your full peer review and any attached files.

If you choose “no”, your identity will remain anonymous but your review may still be made public.

**Do you want your identity to be public for this peer review?**  For information about this choice, including consent withdrawal, please see our Privacy Policy

Reviewer #1: **Yes:** Marin Dujmović

Reviewer #2: **Yes:** Diogenes de Souza Bido

**********

[NOTE: If reviewer comments were submitted as an attachment file, they will be attached to this email and accessible via the submission site. Please log into your account, locate the manuscript record, and check for the action link "View Attachments". If this link does not appear, there are no attachment files.]

To ensure your figures meet our technical requirements, please review our figure guidelines: https://journals.plos.org/plosone/s/figures

You may also use PLOS’s free figure tool, NAAS, to help you prepare publication quality figures: https://journals.plos.org/plosone/s/figures#loc-tools-for-figure-preparation.

NAAS will assess whether your figures meet our technical requirements by comparing each figure against our figure specifications.

# Author comment: Author response to Decision Letter 2

**DOI:** 10.1371/journal.pone.0358575.r004

27 Aug 2026

Editor: PLOS One.

August 27, 2026

Dear Dr Maria José Nogueira;

Thank you very much for considering our revised manuscript, "Psychometric features of the Patient-Centered Communication Scale among Iranian clinical nurses". Manuscript Number: PONE-D-26-05593 for potential placement in PLOS One.

We have revised the manuscript in accordance with the editorial recommendations. Our responses are given in a point-by-point manner in the grid that is presented below. In the revised manuscript, we have highlighted the corrected text in accordance with the reviewers' and editor's recommendations.

We sincerely appreciate the opportunity to provide these revisions to the manuscript and believe the recommendations have continued to improve the paper. We hope the revised version is now suitable for publication and look forward to hearing from you in this regard.

Sincerely,

---

Journal Requirements

Comment: If the reviewer comments include a recommendation to cite specific previously published works, please review and evaluate these publications to determine whether they are relevant and should be cited. There is no requirement to cite these works unless the editor has indicated otherwise.

Response: Thank you for your attention to detail.

The reviewers did not recommend any articles for citation.

---

Comment: Please review your reference list to ensure that it is complete and correct. If you have cited papers that have been retracted, please include the rationale for doing so in the manuscript text, or remove these references and replace them with relevant current references. Any changes to the reference list should be mentioned in the rebuttal letter that accompanies your revised manuscript. If you need to cite a retracted article, indicate the article’s retracted status in the References list and also include a citation and full reference for the retraction notice.

Response: The list of references was re-examined.

While confirming its correctness and completeness, this manuscript does not cite any retracted articles.

---

Additional Editor Comments

Comment: The authors have revised the manuscript in accordance with the recommendations from the previous review round, and the overall quality of the manuscript has improved substantially.

However, several issues identified during the initial review remain insufficiently addressed and require further clarification before the manuscript can be considered for acceptance.

The authors are therefore requested to carefully address the additional comments provided by the reviewers and revise the manuscript accordingly.

Response: Thank you for your encouraging words.

The authors sincerely appreciate the esteemed reviewers and the respected editor for their positive evaluation of the revised manuscript.

We also tried to carefully review all the ambiguities raised and make the necessary corrections in the current version in accordance with the reviewers' suggestions.

---

Reviewer #1

Comment: The authors have responded to comments from myself and the other reviewers thoroughly and I believe the manuscript can get accepted with very minor edits. Thank you for your encouraging words.

Response: It is hoped that the following amendments will enhance the quality of this manuscript.

---

Comment: One suggestion would be to split the data presented in Table 1 so that the two independently drawn samples of 150 nurses can be easily compared rather than collapsed across. They could even be formally compared via stats.

Response: Demographic characteristics are presented separately for the first and second independent samples in Table 1.

These samples were also formally compared on these variables using statistical tests.

In addition, to maintain the sequence of content in both the Materials and methods and the Results, the “Demographic characteristics of participants” section was moved before the “Construct validity” section.

The changes have been highlighted in yellow.

---

Comment: Second, Table 3 shows that the unidimensional model without correlating residuals provided a substantially worse fit than the three-factor model. But then only the unidimensional model was afforded the correlated residuals. I expect nothing would change as the unidimensional solution would remain more parsimonious - but the same affordance should be made for the three-factor model (of course if it was the case that MI-s did not exceed the threshold the authors identified then this is fine).

Response: We sincerely thank the reviewer for this insightful methodological observation.

You are absolutely correct in noting the asymmetry in the use of correlated residuals between the unidimensional and three-factor models.

To clarify: we initially tested the three‑factor model on the second independent sample (n=150) and obtained a satisfactory fit (χ²(39)=104.52, P<0.001, χ²/df=2.68, RMSEA=0.08, CFI=0.98, TLI=0.97, PNFI=0.72, and PCFI=0.73). Because this model already met the conventional fit thresholds, we did not inspect its modification indices for residual covariances, as further adjustments were not required to establish adequate fit.

In contrast, the unidimensional model (without correlated residuals) showed a suboptimal fit (χ²(44)=185.24, P<0.001, χ²/df=4.21, RMSEA=0.12, CFI=0.89, TLI=0.87, PNFI=0.47, and PCFI=0.48). To improve this model, we followed standard CFA practice and examined modification indices, adding only two theoretically justified residual covariances (items 11‑12 and items 6‑7). This brought the unidimensional model to an excellent fit.

The critical comparison between the two structures, however, was based on the final models, the three‑factor model (which already fit well without any residual covariances) and the revised unidimensional model. The chi‑square difference test (Δχ²(3)=4.21, P=0.24) was not significant, and the parsimony indices (AIC, BIC) clearly favored the more parsimonious unidimensional structure. Thus, even if we had examined and added residual covariances to the three‑factor model (which was unnecessary given its already acceptable fit), the outcome of the model comparison would remain unchanged: the unidimensional model is more parsimonious and statistically equivalent in fit.

---

Comment: Third, now that I've revisited the scale, it occurs to me that all of the items are in the same direction and so straight-line responding is a possibility. It is also difficult to control for or identify as it would not be unreasonable to expect the same response on all items.

Response: We sincerely thank the reviewer for this insightful methodological observation.

We fully agree that all items of the Persian version of the PCCS are worded in the same positive direction, which indeed raises the possibility of straight line responding (i.e., respondents uniformly selecting the same Likert option without careful discrimination).

We acknowledge that this is a common limitation in unidimensional, positively framed self report instruments, and it is particularly difficult to detect or control statistically in cross sectional designs. However, we would like to note two points: (a) our sample demonstrated adequate variability in total scores (range 11–55, SD≈14.8), suggesting that not all participants engaged in careless uniform responding; and (b) the excellent model fit and high factor loadings (0.77–0.96) indicate that the items strongly cohere around the latent construct, which somewhat mitigates concerns about random responding.

To address this issue transparently, we have added a brief statement to the Limitations, strengths, and suggestions subsection of the Discussion, acknowledging this potential bias and recommending that future studies consider including reverse scored items or employing careless response detection methods (e.g., longstring analysis, Mahalanobis distance, or response time monitoring) to further strengthen the scale's validity.

We are grateful for this valuable comment, which has improved the methodological rigor of our manuscript.

---

Comment: Fourth, it occurs to me that the mean, median, SD, range and other descriptive parameters for the total scale score were not reported anywhere. I'd suggest doing so for both of the samples only for the unidimensional solution. This is useful data to people considering the scale.

Response: Thank you for your valuable comment.

Descriptive indices such as mean, median, standard deviation (SD), and range (minimum and maximum) for the overall scores of the single-factor structure of this scale were calculated separately for the first and second independent samples.

The changes have been highlighted in yellow.

---

Comment: Finally, in the discussion - is there a reason to assume that relationship status would / should impact this scale? If not, I'd drop that bit. The big difference in work experience however is a good addition. Thank you for your constructive feedback.

Response: The sentences regarding the apparent difference between the marital status of the participants in the current study and the initial study of scale design were removed from the “Discussion”, and only the apparent difference between their work experience was emphasized as one of the possible reasons for the difference in the dimensionality of the original and Persian versions of the Patient-Centered Communication Scale.

The changes have been highlighted in yellow.

---

Reviewer #2

[1] Unifactorial or tri-factorial?

Comment: Ok

Response: Thank you very much.

---

[2] Viewpoint of nurses

Comment: Ok

Response: Thank you very much.

---

[3] normal X “robust to moderate violations of normality”

>> 1st submission - p.13 and 15) “indicated normal univariate and multivariate distribution of the items, respectively.”

>> R1 - p.16) “Skewness values spanning -3.00 to +3.00 and kurtosis values spanning -7.00 to +7.00, as well as Mardia coefficient values less than 8.00, indicated normal univariate and multivariate distribution of the items, respectively.”

Comment: OK, “ML estimation is robust to moderate violations of normality”

But we cannot conclude that these items have a normal distribution.

Suggestion: Remove this sentence or cite methodological authors that justify this statement.

Response: Thank you for your attention to detail.

The phrase “indicated normal univariate and multivariate distribution of the items” was changed to “indicated no substantial deviation from the univariate and multivariate normal distribution of the items”.

The changes have been highlighted in yellow.

In addition, skewness values spanning -3.00 to +3.00 and kurtosis values spanning -7.00 to +7.00, as well as Mardia coefficient values less than 8.00, were also reported based on the following references in the “Normal distribution and outlier values” section of the Materials and methods.

• Sharif-Nia H, Marôco J, Hoseinzadeh E, Moshtagh M, Hatamipour K. Validity and reliability of the Persian version of the gender equity scale in nursing education. BMC Nurs. 2025;24(1):187. doi: 10.1186/s12912-025-02831-5.

• Ghasempour S, Bagheri H, Sharif-Nia H, Marôco J, Miraghai F, Abbasi A. Psychometric characteristics of the Persian version of the State Self-Compassion Scale in patients with cardiovascular diseases. Acta Psychol. 2026;266:106942. doi: 10.1016/j.actpsy.2026.106942.

---

[4] Correlations between the error terms

Comment: Ok

Response: Thank you very much.

---

[5] Persian version as supplementary files.

Comment: Ok

Response: Thank you very much.

Attachment Submitted filename: Response_to_Reviewers_auresp_2.pdf

# Editor report: Decision Letter 2

**DOI:** 10.1371/journal.pone.0358575.r005

**Authors:** Maria José Nogueira

3 Sep 2026

Psychometric features of the Patient-Centered Communication Scale among Iranian clinical nurses

PONE-D-26-05593R2

Dear Dr. Saeed Ghasempour

We’re pleased to inform you that your manuscript has been judged scientifically suitable for publication and will be formally accepted for publication once it meets all outstanding technical requirements.

Within one week, you’ll receive an e-mail detailing the required amendments. When these have been addressed, you’ll receive a formal acceptance letter and your manuscript will be scheduled for publication.

An invoice will be generated when your article is formally accepted. Please note, if your institution has a publishing partnership with PLOS and your article meets the relevant criteria, all or part of your publication costs will be covered. Please make sure your user information is up-to-date by logging into Editorial Manager at Editorial Manager® and clicking the ‘Update My Information' link at the top of the page. For questions related to billing, please contact billing support.

If your institution or institutions have a press office, please notify them about your upcoming paper to help maximize its impact. If they’ll be preparing press materials, please inform our press team as soon as possible -- no later than 48 hours after receiving the formal acceptance. Your manuscript will remain under strict press embargo until 2 pm Eastern Time on the date of publication. For more information, please contact onepress@plos.org.

Kind regards,

Maria José Nogueira, Ph.D.

Academic Editor

PLOS One

Additional Editor Comments (optional):

Dear Authors

The authors responded appropriately to the comments from other reviewers. The manuscript is now ready for acceptance for publication.

Reviewers' comments:

# Editor report: Acceptance letter

**DOI:** 10.1371/journal.pone.0358575.r006

**Authors:** Maria José Nogueira

PONE-D-26-05593R2

PLOS One

Dear Dr. Ghasempour,

I'm pleased to inform you that your manuscript has been deemed suitable for publication in PLOS One. Congratulations! Your manuscript is now being handed over to our production team.

At this stage, our production department will prepare your paper for publication. This includes ensuring the following:

* All references, tables, and figures are properly cited

* All relevant supporting information is included in the manuscript submission,

* There are no issues that prevent the paper from being properly typeset

You will receive further instructions from the production team, including instructions on how to review your proof when it is ready. Please keep in mind that we are working through a large volume of accepted articles, so please give us a few days to review your paper and let you know the next and final steps.

Lastly, if your institution or institutions have a press office, please let them know about your upcoming paper now to help maximize its impact. If they'll be preparing press materials, please inform our press team within the next 48 hours. Your manuscript will remain under strict press embargo until 2 pm Eastern Time on the date of publication. For more information, please contact onepress@plos.org.

You will receive an invoice from PLOS for your publication fee after your manuscript has reached the completed accept phase. If you receive an email requesting payment before acceptance or for any other service, this may be a phishing scheme. Learn how to identify phishing emails and protect your accounts at https://explore.plos.org/phishing.

If we can help with anything else, please email us at customercare@plos.org.

Thank you for submitting your work to PLOS One and supporting open access.

Kind regards,

PLOS One Editorial Office Staff

on behalf of

Professor Maria José Nogueira

Academic Editor

PLOS One
